# Botanical and Geographical Origin Assessment of Olive Oil from Known and Lesser-Known Greek Olive Cultivars Using Mineral Content and Chemometrics

**DOI:** 10.3390/foods15193536

**Published:** 2026-10-03

**Authors:** Eirini Intzirtzi, Eleni C. Mazarakioti, Dimitrios G. Lazaridis, Anastasios Zotos, Angelos Patakas, Athanasios Ladavos, Ioannis K. Karabagias

**Affiliations:** 1 Department of Food Science & Technology, School of Agricultural Sciences, University of Patras, G. Seferi 2, 30100 Agrinio, Greece; up1076528@ac.upatras.gr (E.I.); e.mazarakioti@upnet.gr (E.C.M.); apatakas@upatras.gr (A.P.); alantavo@upatras.gr (A.L.); 2 Department of Sustainable Agriculture, University of Patras, G. Seferi 2, 30100 Agrinio, Greece; azotos@upatras.gr

**Keywords:** olive oil, minerals, ICP-MS, geographical origin, botanical origin, chemometrics

## Abstract

Olive oil is a natural oil that preserves the flavor, aroma, vitamins, and properties of the olive. The most significant challenge in olive oil production is maintaining its stable chemical composition, as oxidation is the primary cause of its deterioration. Olive oil contains minerals in low concentrations, and the presence of high concentrations of minerals contributes to increased oxidation of the oil. The presence of heavy metals in olive oil can be attributed to natural, chemical, and environmental factors. Various analytical techniques are available to determine minerals in olive oil. This study aimed to verify the geographical and botanical origin of olive oil samples collected from various Greek regions using inductively coupled plasma mass spectrometry (ICP-MS). A total of 36 minerals were determined. Regarding geographical origin analysis of olive oil of the Koroneiki olive cultivar, the dominant minerals were Al, B, Ba, Ca, Cr, Cu, Fe, Ga, Li, Mg, Mn, Mo, Na, Ni, Pb, Rb, Se, Sr, W, Zn and Zr, while in the botanical origin analysis (Koroneiki, Ntopia Androu, Adramitiani, Ladoelia, Chondroelia, Ntopia Chalkidikis) the dominant minerals were Al, Cu, Fe, Mn, Na, Ni, Rb, Se, Ti. Significant (*p* < 0.05) variations in mineral content were observed among the samples, suggesting differences owed to geographical and botanical origins. Multivariate statistics, based on multivariate analysis of variance, linear discriminant analysis, and factor analysis, classified samples and provided correlations between minerals and the geographical and botanical origin of olive oil. Classification results based on linear discriminant analysis and using two prior probabilities (all groups are equal or compute from group sizes) during linear discriminant analysis in relation to the cross-validation method showed that mineral content analysis can be used more effectively for the determination of olive oil geographical origin (correct classification rate of 76.9% and 74.4%, respectively), whereas its botanical origin (correct classification rate of 50–56.8%) can be affected by intrinsic or extrinsic factors.

## 1. Introduction

Olive oil is one of the most valuable products in the Mediterranean diet, known for its medicinal properties, as this can be confirmed by various clinical and experimental studies in the literature [1]. Moreover, olive oil is important for its nutritional benefits, as it is rich in monounsaturated fatty acids, antioxidants, and phenolic compounds; contamination with heavy metals can have not only qualitative and economic but also health implications [2].

Since the beginning of the Industrial Revolution, environmental pollution from toxic metals has increased. Although the soil contains an amount of heavy metals, the contamination occurs mainly from pollution by local sources. Such sources include industries, particularly those that produce iron and steel, and those from the chemical industries. Another significant source of pollution is the use of contaminated irrigation water, the use of pesticides, the use of phosphate-rich fertilizers, and the use of contaminated manure. Furthermore, sewage incineration and the burning of fossil fuels also leave a toxic environmental footprint [3]. As a result of the above actions, food contamination has increased.

In edible oils and olive fruit, the presence of metals may be due to various factors, such as their natural presence, their introduction during the refining process, and their contact with metallic storage materials. More extensively, the minerals in olive fruit are important from both a nutritional and a toxicological perspective. Some elements, including Fe, Cu and Zn, are essential micronutrients, while inadequate intake may be associated with adverse health effects [4].

Additionally, trace elements (Fe, Cu, Ca, Mg, Co, Ni, Mn, As, Cr, Cd, and Pb) can affect the shelf-life and freshness of olive oils as they act as catalysts for oxidative reactions involving aldehydes, ketones, and acids. For example, traces of Cu (0.1–1 μg g^−1^) and Fe (1 μg g^−1^) cause rancidity in fats, as they act as catalysts for the oxidation of unsaturated bonds in lipids, leading to rapid spoilage [5]. The natural presence of metals in olive oil can occur through the bioaccumulation of naturally occurring metals from the soil, as well as through environmental pollution [6]. Specific heavy metals such as As, Cr and Cd are considered important contaminants in food due to their toxicity [3]. Their persistence in the environment and prolonged exposure risks to human health, as these minerals can interact with biological components and contribute to adverse effects, including damage to the digestive system and an increased risk of cancer [7,8]. Researchers have used the mineral content of olive oil to characterize not only its geographical origin but also its botanical origin [9]. The multielemental composition of olive oil is a powerful marker of its geographical origin and a useful tool for combating food fraud [10,11]. Various analytical techniques have been employed for olive oil authentication, including high-performance liquid chromatography (HPLC), inductively coupled plasma atomic emission spectrometry (ICP-AES), inductively coupled plasma mass spectrometry (ICP-MS), and other analytical approaches [12]. ICP-MS represents a powerful analytical approach for multielement determination in complex food matrices. ICP-MS enables the accurate quantification of microelements, macroelements, and potential toxic elements [13]. In addition, chemometric tools, such as principal component analysis (PCA) and linear discriminant analysis (LDA) are widely used to process complex analytical data and enhance the discrimination and classification of olive oils according to their geographical and botanical origin [12]. Despite the growing interest in olive oil authentication, a significant gap has been identified in the literature regarding the elemental characterization of Greek olive oils. While numerous studies have focused on the physicochemical properties, volatile compounds, fatty acid composition and other quality characteristics of olive oil [14,15,16,17], relatively limited attention has been paid to its elemental composition. The determination of the geographical origin of olive oil is of major importance for authenticity and traceability purposes. However, its accurate classification requires the consideration of multiple factors. Environmental factors, including climate, soil composition, and geographical location, can affect the content and structure of bioactive components in food matrices [18]. Each production region differs in terms of climatic and geological conditions, which directly influence the characteristics of both the olive fruit and the resulting olive oil.

Several Greek olive varieties have not been sufficiently studied, particularly with regard to their mineral profiles. Therefore, the selection of the geographical regions and olive varieties in the present study aimed to provide new information on the effect of different environmental and climatic conditions on the mineral profile of the same variety, as well as on the potential to distinguish between varieties based on the mineral composition.

The purpose of the present study was the geographical and botanical origin classification of olive oils from various regions of Greece (Aitoloakarnania, Messinia, Lesvos, Andros, Achaia, Fthiotida, Chalkidiki, Crete, Kozani, Thesprotia, Argolida, and Evia) and from different cultivars of known and lesser-known olive cultivars, such as Ntopia Androu, based on the combination of multivariate statistical analyses, including multivariate analysis of variance, linear discriminant analysis, and factor analysis. To our knowledge, only a limited number of studies have focused on specific minerals that are associated with the authenticity of olive oil from different geographical regions and olive cultivars; this comprises the novelty of the present study. In addition, the study provides analytical data regarding the topography and climate conditions in the areas of olive oil production and how these may affect the overall classification results.

## 2. Climate and Olive Oil

Climate is one of the most important physiogeographical factors affecting plant growth and development [17]. Climate change threatens the olive agroecosystem, a crop that is resistant to drought. According to studies, the flowering of olive trees is greatly influenced by spring temperatures, which have shown an increase over time. Olive oil composition is affected by several factors, including genetic factors, cultivation practices, the age of the olive trees, the fruit maturity at harvest, and geographical and climatic conditions [19,20]. High temperatures during flowering, dry summers, and intense solar radiation have led to reduced fruit production and a decline in olive oil quality. Greece is characterized by high temperatures and low rainfall, particularly during the summer months, which causes severe heat stress, sun damage, and tissue necrosis in plants. These factors pose a risk to the survival of olive trees. Olive trees are commonly cultivated under rainfed conditions in many regions. The decline in water availability and rainfall observed in recent years has negatively affected both olive yield and oil quality [21,22]. The annual rainfall requirement of olive trees is 600–800 mm [17]. Furthermore, atmospheric humidity significantly affects olive cultivation. Specifically, in areas where atmospheric humidity is <30% during the olive flowering period, flower drop may occur, leading to a decrease in production. During the fruit development period, a lack of irrigation can cause the fruit to shrink, primarily resulting in a decline in its quality characteristics and a reduction in the production of olive oil or table olives. If the atmospheric humidity level is >70%, it has a negative effect, resulting in the appearance of fungal diseases that are difficult to control [23]. The amount of soil water required by olive trees is more than 450 mm. Soil moisture saturation < 10 cb causes root system suffocation and reduces nutrient uptake. Sudden changes in soil moisture can induce stress in olive trees, resulting in a loss of flowers or fruits. Drought conditions > 25 cb are particularly dangerous for olive cultivation. During fruit ripening, drought leads to the deterioration of both the quality and quantity of the final product [24]. According to the literature, there is a strong relationship between soil and climatic factors and olive oil quality. More specifically, Hemida et al. [25] demonstrated that climatic and soil conditions are responsible for the high quality of olive oil [26]. The adaptation of olive trees to climatic conditions requires an adequate supply of mineral nutrients. The mineral nutrient status of olive trees reflects their condition with respect to nutrient depletion and availability. According to previous studies, the stress experienced by olive trees during periods of drought did not affect the efficiency of mineral nutrient utilization [27]. Trace elements are naturally present in soils at different concentrations and can be taken up by plants through their root systems. Consequently, the mineral profile of plant tissues can reflect the characteristics of the soil. The geographical composition of the soil, which varies from region to region, is transferred to the olive oil. Elements can be introduced into the soil through geochemical changes in rocks, as well as through human activities, such as the use of fertilizers and pesticides containing metals [28]. The geographical composition of the soil, which varies from region to region, is transferred to the olive oil. As a result, the levels of chemical elements in olive oil depend on the specific olive-growing region. This fact allows us to distinguish olive oils from different geographical regions. In addition, the concentrations of chemical elements in olive oils are influenced by their geographical origin [10]. In this context, Supplementary Tables S1 and S2 provide the meteorological stations, the geographical location and topography, and climate conditions of the regions where olive oil samples were produced [29] for additional evaluation of data.

## 3. Materials and Methods

### 3.1. Olive Oil Samples

**Figure 1:**
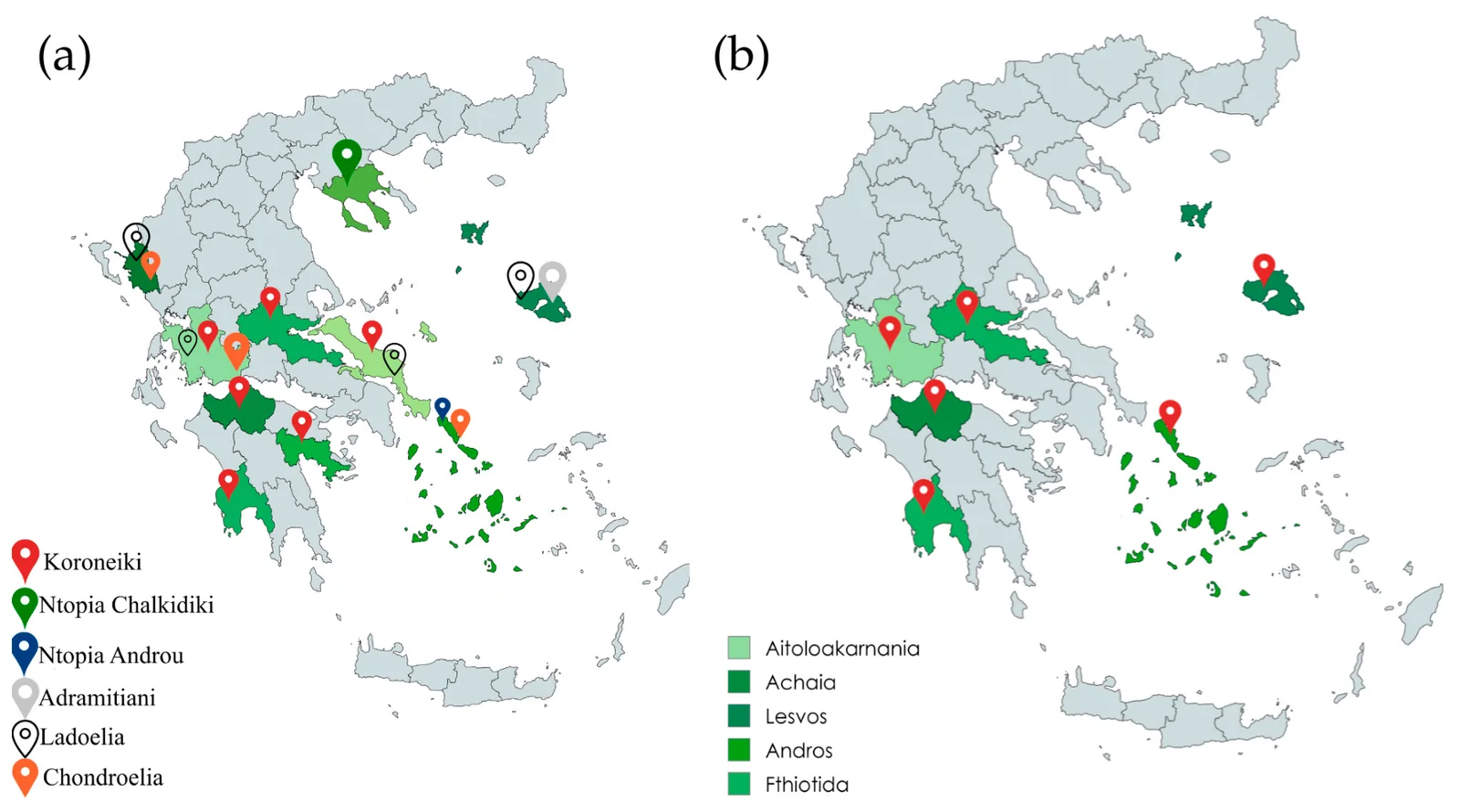
(**a**) Olive cultivars of which olive oil was collected and (**b**) the relative geographical areas where these cultivars are grown.

The olive oil samples used were produced between November 2024 and May 2025 by local olive oil producers from various geographical regions, corresponding to the harvest season (2024–2025). Specifically, 42 olive oil samples were collected for geographical classification from the following regions: Aitoloakarnania (*N* = 15), Andros (*N* = 6), Messinia (*N* = 13), Fthiotida (*N* = 5), and Achaia (*N* = 3). A total of 88 olive oil samples were used for the botanical origin identification, representing olive varieties including Koroneiki (*N* = 45), Ntopia Androu (*N* = 15), Adramitiani (*N* = 7), Ladoelia (*N* = 5), Chondroelia (*N* = 8), and Ntopia Chalkidikis (*N* = 8). All samples were stored in Falcon tubes in a refrigerator at 4 ± 1 °C from the day of their arrival at the laboratory until the day of analysis. Figure 1 indicates the geographical areas and the relative olive cultivars where olive oil samples were collected.

### 3.2. Reagents, Standards for Mineral Content Analysis (ICP-MS)

Highly pure nitric acid (70% *v*/*v* TraceSelect, for trace analysis) was obtained from Honeywell Fluka (Seelze, Germany), while the hydrogen peroxide solution (H_2_O_2_ 30% w/w, for ultra-trace analysis) was supplied by Sigma-Aldrich (Darmstadt, Germany). The calibration standard solutions A (100 mL: 10 μg/mL of Ag, Al, As, Ba, Be, Ca, Cd, Co, Cr, Cs, Cu, Fe, Ga, K, Li, Mg, Mn, Na, Ni, Pb, Rb, Se, Sr, Tl, U, V, Zn in a matrix of 5% HNO_3_), B (10 μg/mL B, Ge, Mo, Nb, P, Re, S, Si, Ta, Ti, W, Zr in a matrix of 0.2% HF, and traces of HNO_3_), Rh ICP internal standard solution (100 mL: 10 μg/mL in a matrix of 2% HCl), and the turning solution (500 mL: 10 μg/ mL Li, Y, Ce, Tl, and Co in a matrix of 5% HNO_3_) were purchased from Agilent (Santa Clara, CA, USA).

### 3.3. Instrumentation

The contents of 36 minerals (Li, Be, B, Na, Mg, Al, K, Ca, Ti, V, Cr, Mn, Fe, Co, Ni, Cu, Zn, Ga, Ge, As, Se, Rb, Sr, Zr, Nb, Mo, Ag, Cd, Cs, Ba, Ta, W, Re, Tl, Pb, U) were measured using a quadrupole ICP-MS, Agilent Technologies 7850 series model (Santa Clara, CA, USA). In the case of Pb, all three isotopes of Pb (^206^Pb, ^207^Pb, and ^208^Pb) were monitored to reduce possible interferences. For data extraction, MassHunter 5.1 software (Agilent Technologies) was employed. The digestion procedure of the olive oil samples was carried out using the microwave oven Multiwave Go Plus (Anton Paar GmbH, Graz, Austria). Ultrapure water suitable for trace element analysis was obtained by the Smart2Pure Water Purification System (Thermo Electron LED GmbH, Langenselbold, Germany). The operating conditions are given in Supplementary Table S3.

### 3.4. Determination of Mineral Content and Analytical Validation Parameters

Sample digestion was carried out according to the procedure reported by the method of Dalla et al. [30]. Specifically, 0.250 g of each olive oil sample was transferred into Teflon vessels and digested with 4 mL of pure HNO_3_ (70%) and 1 mL of H_2_O_2_ (30%) in a microwave oven under specific conditions. The resulting digested samples were greenish and clear solutions, which were transferred into Falcon tubes and diluted to a final volume of 50 mL with 18.2 MΩ water. The final solutions were stored under refrigeration at 4 ± 1 °C. All the reusable containers during the experimental procedure were acid-washed with diluted HNO_3_ in ultra-pure water before their use to avoid any contamination. Supplementary Tables S4 and S5, and Supplementary Figure S1, provide the analytical validation parameters, including recovery rates based on reference material, limits of detection and quantification, along with the calibration curves of the analyzed elements.

### 3.5. Statistical Analysis

The chemometric evaluation of data included both supervised and unsupervised statistical techniques such as multivariate analysis of variance (MANOVA) and linear discriminant analysis (LDA) along with factor analysis [30]. All variables were considered significant at the confidence level *p* < 0.05. During the LDA, two prior probabilities were investigated: (a) all groups are equal, and (b) compute from group sizes, given that the number of olive oil samples that were collected was different. In both cases, only the significant (*p* < 0.05) variables were introduced in the model. In addition, the raw data were used in statistical analysis to mimic real analysis conditions. The confidence interval of LDA was evaluated using the cross-validation method [30]. For mean separation and comparison of pairs of individual groups, Tukey’s post hoc test was used. The statistical analysis of data was carried out using the SPSS software v. 28.0 (IBM Inc., 2021, Armonk, NY, USA).

## 4. Results

### 4.1. Elements Below the Detection Limit

The analysis revealed that several elements were either not identified or were present only at trace concentrations in the examined olive oil samples. Of the 36 metals identified in the olive oil samples for their geographical classification, K, V, Co, Ge, As, Nb, Ag, Cd, Cs, Ta, Re, Ti, U, and Be were generally below the detection limit. The elements that were below detection limits and were detected only in a limited number of samples were excluded from the statistical analysis and were reported descriptively. However, trace concentrations of some of these elements were found in a limited number of samples. In Aitoloakarnania, V (0.001–0.002 μg/L), Co (0.001 μg/L), Ag (0.004–0.039 μg/L), Cd (0.001–0.002 μg/L), Cs (0.002 μg/L), Ta (0.008–0.005 μg/L), Re (0.002 μg/L), Ti (0.001 μg/L), U (0.001–0.002 μg/L), Be (0.003–0.006 μg/L). In Fthiotida, amounts of V (0.001 μg/L), Ag (0.003 μg/L), and Cd (0.001–0.002 μg/L) were identified. Ag was also identified in one olive oil sample from the Messinia region (0.001 μg/L). For the botanical origin classification of olive oil samples, Be, K, V, Co, As, Nb, Ag, Cd, Cs, Ta, Re, Ti, U, and Ge were generally below the detection limit. The Koroneiki variety contained detectable levels of Be (0.003–0.006 μg/L), V (0.001–0.002 μg/L), Co (0.001 μg/L), Nb (0.001–0.014 μg/L), Ag (0.003–0.039 μg/L), Cd (0.001–0.008 μg/L), Cs (0.002 μg/L), Ta (0.005–0.008 μg/L), Re (0.002 μg/L), Ti (0.001 μg/L), U (0.001–0.002 μg/L), Ge (0.001 μg/L). In the Adramitiani olive cultivar, V (0.006–0.003 μg/L) and As (0.002 μg/L) were identified. The same holds for the Ladoelia olive cultivar, where V (0.004–0.007 μg/L) and As (0.002 μg/L) were identified in relatively similar amounts. Similarly, in the Chondroelia olive cultivar, Nb (0.001 μg/L) was identified. Finally, in the Ntopia Chalkidikis cultivar, Ag (0.002 μg/L), Cd (0.002 μg/L), and Ge (0.001 μg/L) were identified.

### 4.2. Mineral Content of Olive Oil According to Geographical Origin

For the geographical origin classification of olive oil samples, Li, B, Na, Al, Cr, Mo, W, and Se did not show statistically significant (*p* > 0.05) differences among the regions (as shown in Table 1). In contrast, statistically significant *(p* < 0.05) regional differences in olive oil samples were observed for Mg, Ca, Mn, Fe, Ni, Cu, Ga, Rb, Sr, Ba, and Pb. Fthiotida showed the highest values for Mg (1.56 ± 0.87 μg/L), Mn (0.08 ± 0.04 μg/L), Fe (2.40 ± 0.59 μg/L), Ni (0.07 ± 0.07 μg/L), Cu (0.24 ± 0.06 μg/L), Ga (0.18 ± 0.01 μg/L), Sr (0.10 ± 0.03 μg/L), and Ba (0.13 ± 0.04 μg/L) and the lowest were shown in Messinia (0.05 ± 0.09, 0.01 ± 0.02, 0.44 ± 0.21, 0.00 ± 0.00, 0.02 ± 0.02, 0.00 ± 0.00, 0.00 ± 0.00, and 0.00 ± 0.00 μg/L, respectively). Similarly, Ca reached the highest content in Andros (2.00 ± 1.83 μg/L) and the lowest in Messinia (0.01 ± 0.02 μg/L). Titanium content was the highest in the Aitoloakarnania region (0.05 ± 0.06 μg/L), whereas the Andros, Messinia, and Achaia regions showed values of 0.00 ± 0.00 μg/L. Finally, regarding Pb, the highest content (0.22 ± 0.35 μg/L) was recorded in the Andros region and the lowest in the Messinia (0.00 ± 0.01 μg/L) region. Messinia showed statistically significant (*p* < 0.05) differences in mineral content compared with most of the other geographical regions. The Fthiotida region showed statistically significant (*p* < 0.05) differences in Pb content compared to the other regions.

### 4.3. Mineral Content of Olive Oil According to Olive Cultivar

For the classification of olive oil samples according to olive cultivar, Mn, Fe, Cu, Se, Rb, Na, Al, Ti, and Ni showed statistically significant (*p* < 0.05) differences (Table 2). Manganese content was the highest in Ladoelia cultivar (0.05 ± 0.06 μg/L) and the lowest in Ntopia Androu (0.00 ± 0.00 μg/L), with statistically significant (*p* < 0.05) differences between the two cultivars. Similarly, Fe reached the highest content in the Ladoelia cultivar (4.69 ± 3.29 μg/L) and the lowest in the Koroneiki cultivar (1.11 ± 1.09 μg/L). The Ladoelia cultivar showed significant (*p* < 0.05) differences from all the other cultivars. The Adramitiani cultivar also showed statistically significant (*p* < 0.05) differences from the Ntopia, Andriotiki, Koroneiki, and Chondroelia cultivars. Copper content was the highest in the Ladoelia cultivar (0.36 ± 0.21 μg/L) and the lowest in the Chondroelia and Koroneiki cultivars (0.13 ± 0.08 and 0.13 ± 0.10 μg/L, respectively). The Koroneiki cultivar varied significantly (*p* < 0.05) in Cu content compared to the Ladoelia, Adramitiani (0.33 ± 0.31 μg/L) and Ntopia Chalkidikis (0.30 ± 0.11 μg/L) cultivars. For Se, the highest content was recorded in the Chondroelia cultivar (0.01 ± 0.01 μg/L) and showed statistically significant (*p* < 0.05) differences compared to the other cultivars. Rubidium varied significantly (*p* < 0.05) between Adramitiani (0.00 ± 0.01 μg/L) and Koroneiki cultivars, Ntopia Androu, as well as Chondroelia and Ntopia Chalkidikis (0.00 ± 0.00 μg/L) cultivars. Concerning Na, the highest content was recorded in the Ladoelia (8.03 ± 5.45 μg/L) cultivar, and the lowest in Ntopia Androu (0.12 ± 0.48 μg/L). Sodium content resulted in statistically significant (*p* < 0.05) differences in the Ladoelia cultivar among Ntopia Androu, Koroneiki (1.19 ± 2.20 μg/L), Chondroelia (1.26 ± 1.89 μg/L), and Ntopia Chalkidikis (1.16 ± 2.16 μg/L) cultivars. Aluminum content was also the highest in the Ladoelia (13.39 ± 6.95 μg/L) cultivar and the lowest in the Ntopia Chalkidikis (2.87 ± 1.58 μg/L) cultivar. Finally, for Ti and Ni, the highest content was recorded in Ladoelia (0.07 ± 0.04, 0.07 ± 0.05 μg/L) cultivar and the lowest in Ntopia Chalkidikis and Ntopia Androu (0.00 ± 0.00, 0.01 ± 0.02 μg/L) cultivars, respectively.

## 5. Geographical Origin Classification of Olive Oil

### 5.1. Multivariate Tests

Four qualitative criteria of the multivariate hypothesis during MANOVA, namely Pillai’s Trace = 3.119 (F = 3.056, df = 88, *p* < 0.001), Wilks’ Lambda = 0.000 (F = 4.665, df = 88, *p* < 0.001), Hotelling’s trace = 39.269 (F = 6.470, df = 88, *p* < 0.001), and Roy’s largest root (F = 24.467, df = 22, *p* < 0.001), all having an observed power of 1.000, showed that there was a significant (*p* < 0.05) effect of the cultivar of olive oil on the mineral content of olive oils. The minerals that showed significant (*p* < 0.05) differences among olive oil samples of different geographical origin (Aitoloakarnania, Andros, Messinia, Fthiotida, and Achaia) are listed in Table 1, with 13 out of 22 determined.

### 5.2. Factor Analysis

**Figure 2:**
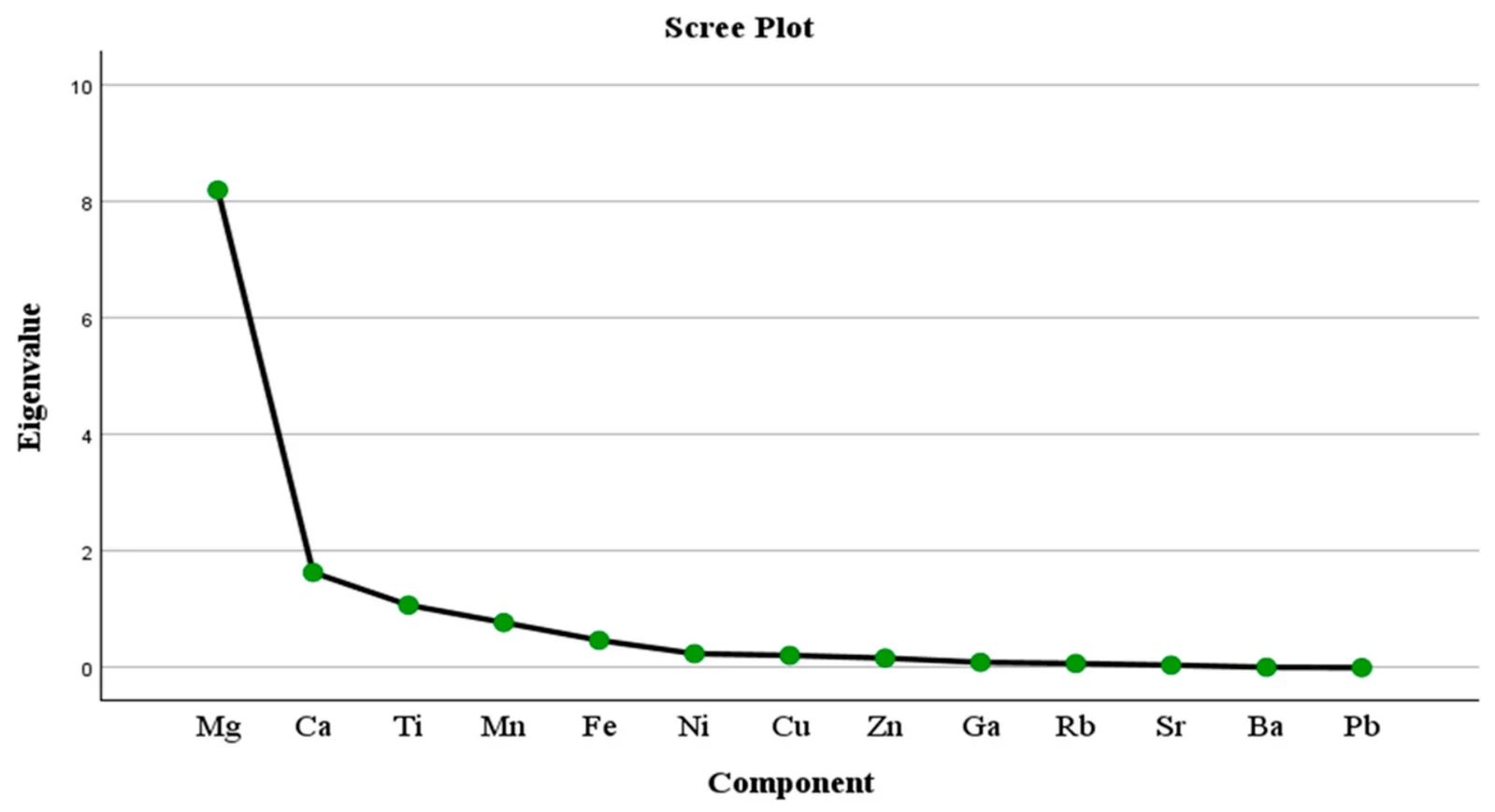
Significant minerals related to olive oil of the Koroneiki olive cultivar in the multidimensional space of factor analysis.

Factor analysis showed that the significant minerals of different olive oil cultivars could explain the variability in the multidimensional space. The Kaiser–Meyer–Olkin (KMO) index was 0.779, while Bartlett’s Test of Sphericity yielded Chi-Square (X^2^) = 766.016, df = 78, *p* < 0.001, indicating the presence of correlations among the significant minerals in relation to olive oil cultivar, thereby allowing the effective application of factor analysis. Figure 2 shows the scree plot of the significant minerals related to the Koroneiki olive oil cultivar of different geographical origins.

The principal components (PCs) that showed the highest correlation (factors) in the rotated space of factor analysis during Varimax rotation were three. Based on the first three PCs, the variance explained was 83.45%, which was considered satisfactory. The mineral content for which the correlation in the rotated component matrix of the multidimensional space was the largest were Sr (PC1, correlation of 0.886 and explained variance of 35.70%), Zn (PC2, correlation of 0.889 and explained variance of 26.92%), and Ti (PC3, correlation of 0.911 and explained variance of 21.23%).

### 5.3. Discrimination of Olive Oil of the Koroneiki Olive Cultivar from Aitoloakarnania, Andros, Messinia, Fthiotida, and Achaia Regions

Four discriminant functions were formed during LDA, even though three were significant (*p* < 0.05) for the classification of olive oil samples according to geographical origin. The analytical data of these discriminant functions, including the Wilks’ Lambda, Chi-square, eigenvalues, explained and cumulative variance, along with canonical correlation, are given in Table 3.

**Figure 3:**
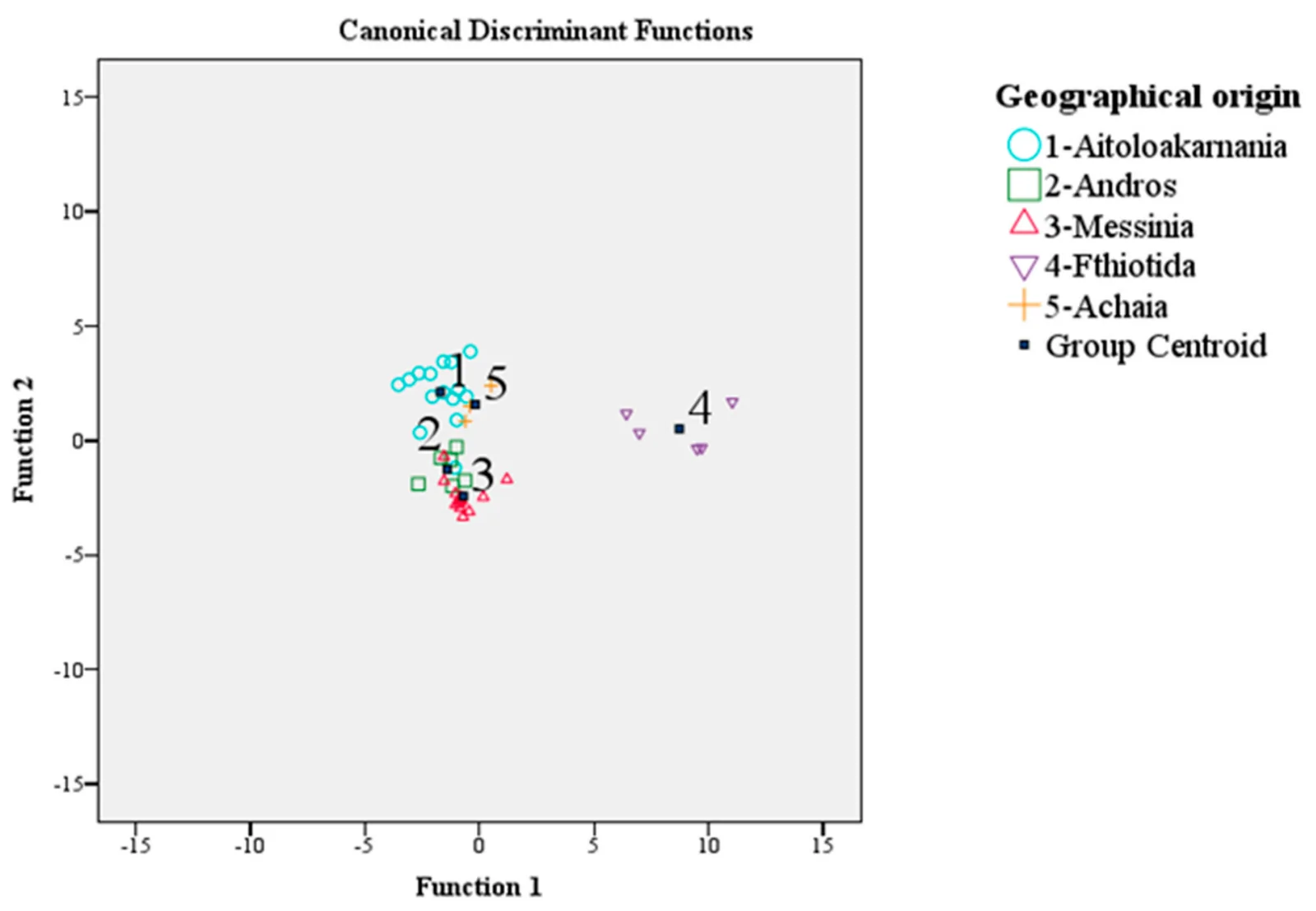
Classification of olive oil of the Koroneiki olive cultivar from Aitoloakarnania, Andros, Messinia, Fthiotida, and Achaia regions based on the significant minerals and LDA.

The overall correct classification rate was 90.5% for the original classification, while the cross-validation method resulted in a rate of 69%. The group centroid values were (−1.702, 2.120), (−1.403, −1.252), (−0.705, −2.428), (8.730, 0.508), and (−0.177, 1.578) for olive oil samples from Aitoloakarnania, Andros, Messinia, Fthiotida, and Achaia regions, respectively (Figure 3).

Moving deeper into the analysis, based on the cross-validation method, of the initial 15 olive oil samples from Aitoloakarnania, 11 were correctly classified, whereas 1 sample was allocated to Andros, 1 sample to Messinia, and 2 samples to Achaia. Similarly, of the initial six olive oil samples from Andros, one was correctly classified, whereas one sample was allocated to Aitoloakarnania, and four samples to Messinia. Regarding the olive oil samples from Messinia, of the initial 13 samples, 12 were correctly classified, and 1 sample was allocated to Aitoloakarnania. Of the five initial olive oil samples from Fthiotida, four were correctly classified, whereas one sample was allocated to Achaia. Finally, of the three initial olive oil samples from Achaia, one was correctly classified, whereas two samples were allocated to Aitoloakarnania.

The analytical results are shown in Table 4. The best classification results were obtained for olive oil samples from Messinia (92.3%), followed by Fthiotida (80%) and Aitoloakarnania (73.3%). Poor results were obtained for olive oil samples from Andros (16.7%) and Achaia (33.3%) regions.

The minerals that contributed most to the classification of olive oil samples from the Koroneiki olive cultivar were those with the highest absolute correlation values in the significant (*p* < 0.05) discriminant functions. These values are presented in bold in Table 5.

### 5.4. Classification of Olive Oil of the Koroneiki Olive Cultivar from Aitoloakarnania, Andros, Messinia, and Fthiotida Regions

Given the limited number of olive oil samples from the Achaia region, these were excluded, and the LDA analysis was repeated in order to investigate whether the classification results of olive oil samples of the Koroneiki olive cultivar according to geographical origin could be improved. In this case, three discriminant functions were formed (Table 6). Indeed, the overall classification rate was improved in both cases: 94.9% for the original and 76.9% for the cross-validation method.

The minerals that contributed most to the classification of olive oil samples of the Koroneiki olive cultivar were those with the highest absolute correlation values within the significant (*p* < 0.05) discriminant functions. These are summarized in bold in Table 7.

**Figure 4:**
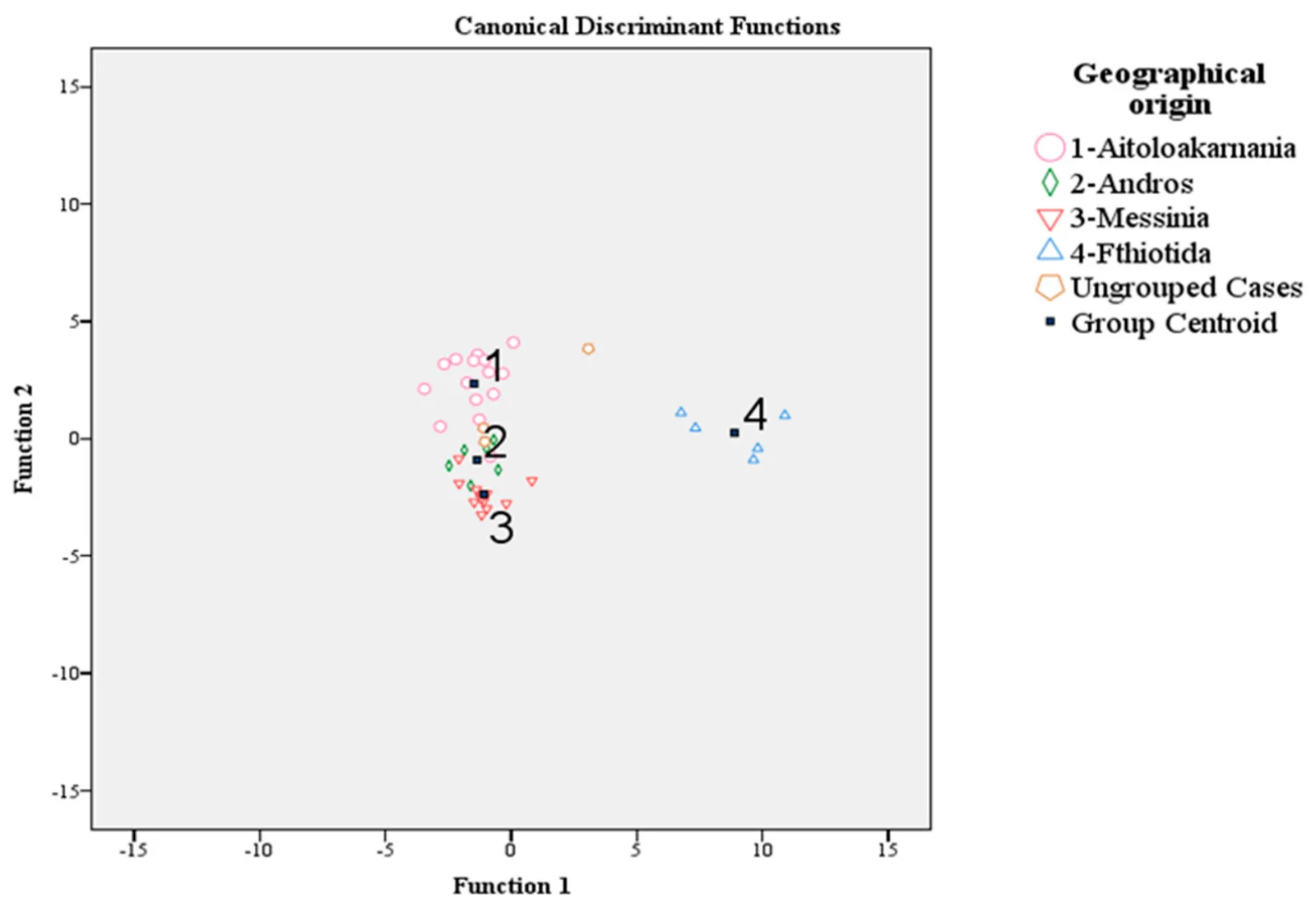
Classification of olive oil of the Koroneiki olive cultivar from Aitoloakarnania, Andros, Messinia, and Fthiotida regions based on the significant minerals and LDA (considering that all groups are equal).

The group centroid values were: (−1.475, 2.343), (−1.539, −0.908), (−1.087, −2.378), and (8.882, 0.244) for olive oil samples from Aitoloakarnania, Andros, Messinia, and Fthiotida regions, respectively (Figure 4). The ungrouped cases in Figure 4 refer to the olive oil samples from Achaia that were excluded from the analysis. Excluding the olive oil samples from Achaia, a perfect classification (100%) of samples from Messinia was obtained, and the classification of samples from Andros was improved (increased by over 16%) (Table 8).

**Figure 5:**
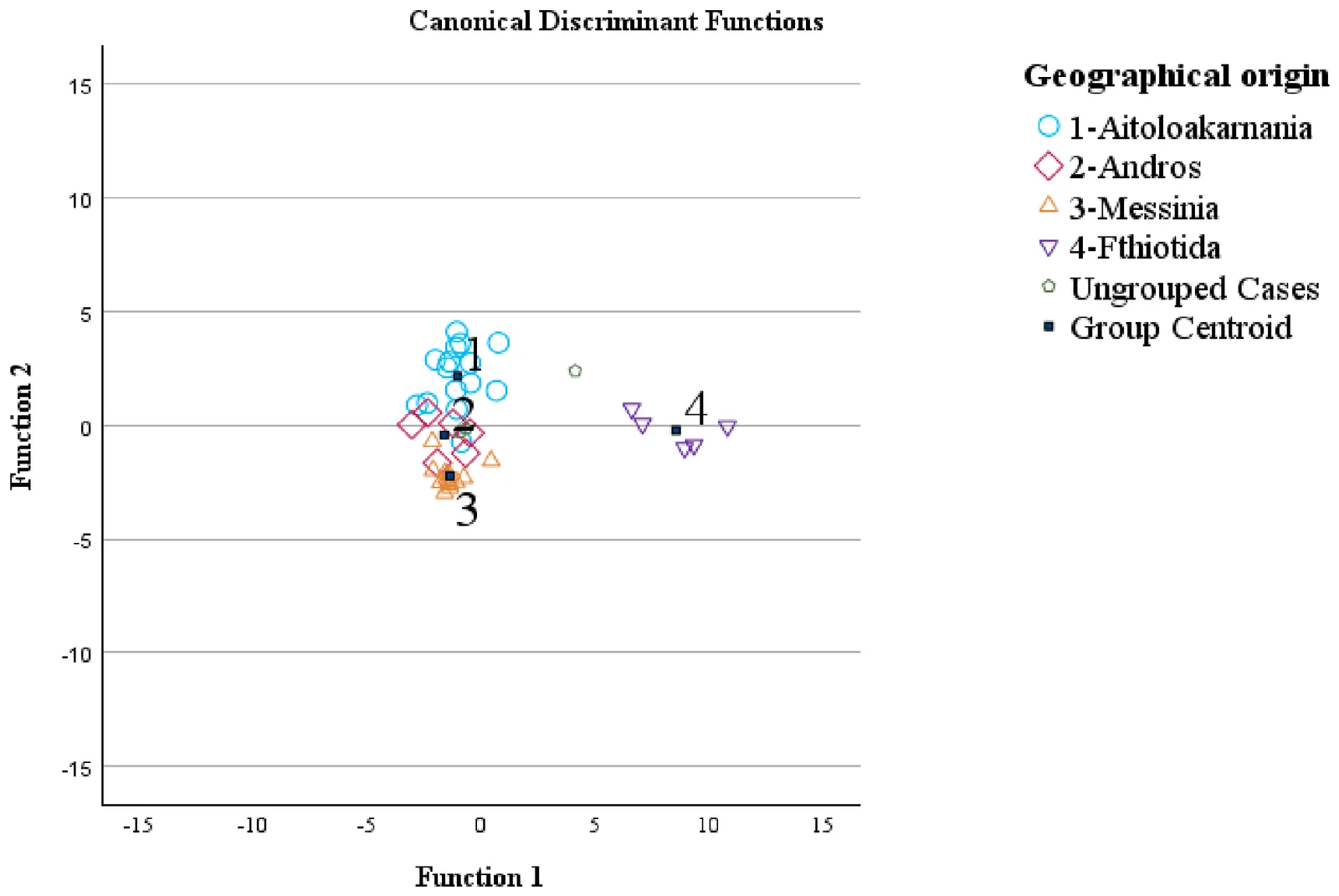
Classification of olive oil of the Koroneiki olive cultivar from Aitoloakarnania, Andros, Messinia, and Fthiotida regions based on the significant minerals and LDA (compute from group sizes).

Considering the effect of the olive oil sample size, LDA was run again, and the classification results are shown in Figure 5 and Supplementary Table S6. Comparing the two prior probabilities in the LDA analysis [(a) all groups are equal or (b) compute from group sizes], computation from group sizes provided slightly lower overall classification rates: 92.3% for the original and 74.4% for the cross-validation method. The classification performance of olive samples from Aitoloakarnania, Messinia, and Fthiotida remained unaffected, whereas the classification rate of olive oil from samples was 16.7% (Supplementary Table S6).

## 6. Classification of Olive Oil According to Olive Cultivar

### 6.1. Multivariate Tests

Four qualitative criteria of the multivariate hypothesis during MANOVA, named Pillai’s Trace = 1.716 (F = 3.056, df = 110, *p* < 0.001), Wilks’ Lambda = 0.083 (F = 1.824, df = 110, *p* < 0.001), Hotelling’s trace = 3.605 (F = 1.947, df = 110, *p* < 0.001), and Roy’s largest root (F = 1.767, df = 22, *p* < 0.001), all having an observed power of 1.000, showed that there was a significant (*p* < 0.05) effect of the cultivar of olive oil on the mineral content of olive oils. Each F-value tests the multivariate effect of olive oil cultivar. These tests are based on the pairwise comparisons among the estimated marginal means that are linearly independent. The minerals that showed significant (*p* < 0.05) differences among olive oil samples of different olive cultivars (Koroneiki, Ntopia Androu, Adramitiani, Ladoelia, Chondroelia, and Ntopia Chalkidikis) are listed in Table 4, and there were 9 (Na, Fe, Cu, Mn, Ni, Al, Ti, Se, and Rb) out of 22 determined.

### 6.2. Factor Analysis

Factor analysis showed that the significant minerals of different olive oil cultivars could explain the variability in the multidimensional space. The Kaiser–Meyer–Olkin (KMO) index, a measure of sampling adequacy, was 0.879 (should be higher than 0.50), while Bartlett’s Test of Sphericity yielded Chi-Square (X^2^) = 437.922, df = 36, *p* < 0.001, indicating the presence of correlations among the significant minerals in relation to olive oil cultivars, thereby allowing the effective application of factor analysis.

**Figure 6:**
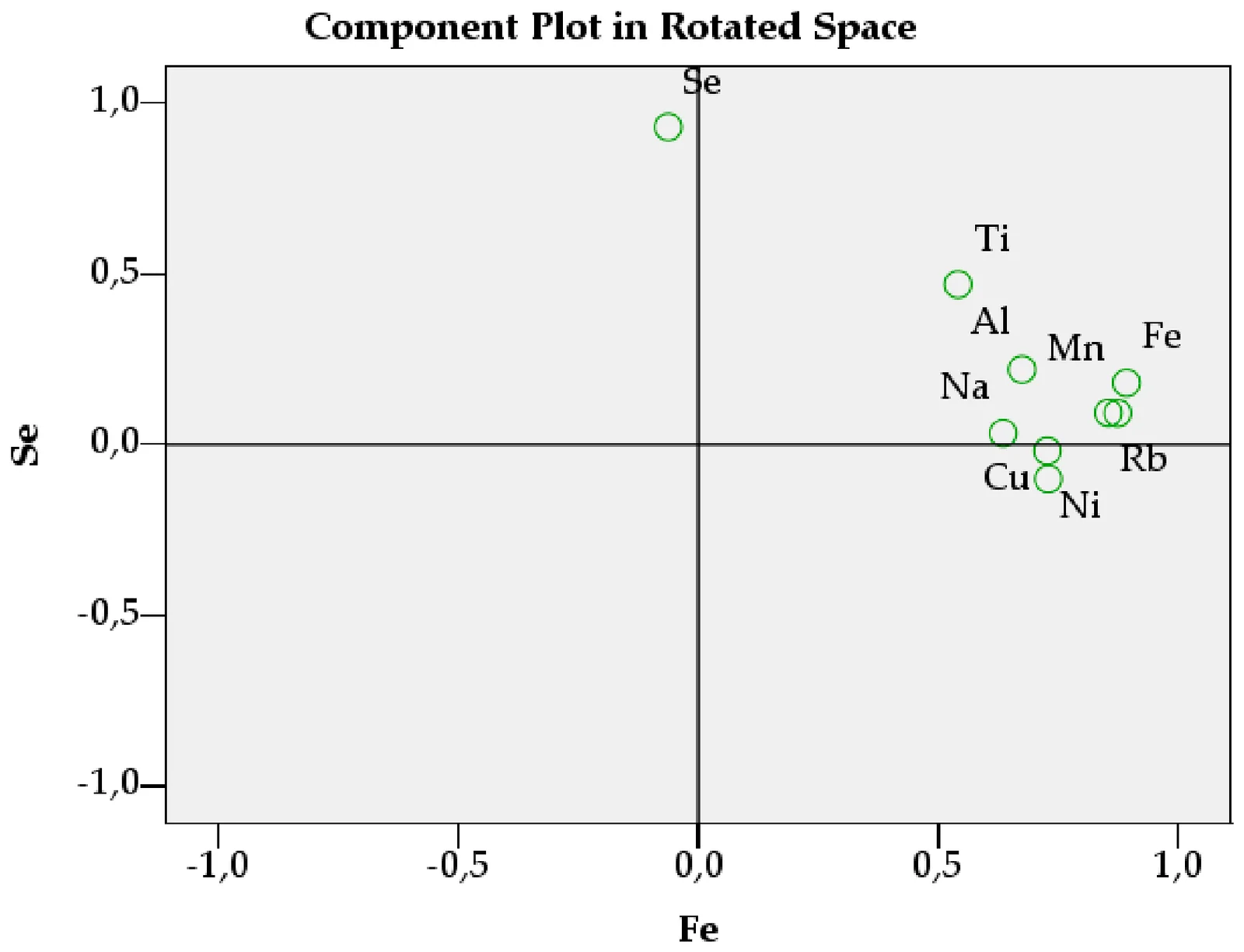
Significant minerals related to olive oil of the different olive cultivars studied, in the multidimensional space of factor analysis.

Figure 6 shows the plot of the significant minerals associated with the olive oil cultivars from the different olive oil cultivars studied.

The principal components (PCs) that showed the highest correlation (factors) in the rotated space of factor analysis during Varimax rotation were two. Based on the first two PCs, the variance explained was 63.42%, considered acceptable. The minerals for which the correlation in the rotated component matrix of the multidimensional space was the largest were Fe (PC1, correlation of 0.893 and explained variance of 50.13%) and Se (PC2, correlation of 0.930 and explained variance of 13.29%).

### 6.3. Classification of Olive Oil of Different Olive Cultivars Based on the Significant Minerals and LDA

Five discriminant functions were obtained during LDA, although three were significant (*p* < 0.05) for the classification of olive oil samples according to olive cultivar. Table 9 presents the analytical results of these discriminant functions, including Wilks’ Lambda, Chi-square, eigenvalues, explained and cumulative variance, and canonical correlation.

**Figure 7:**
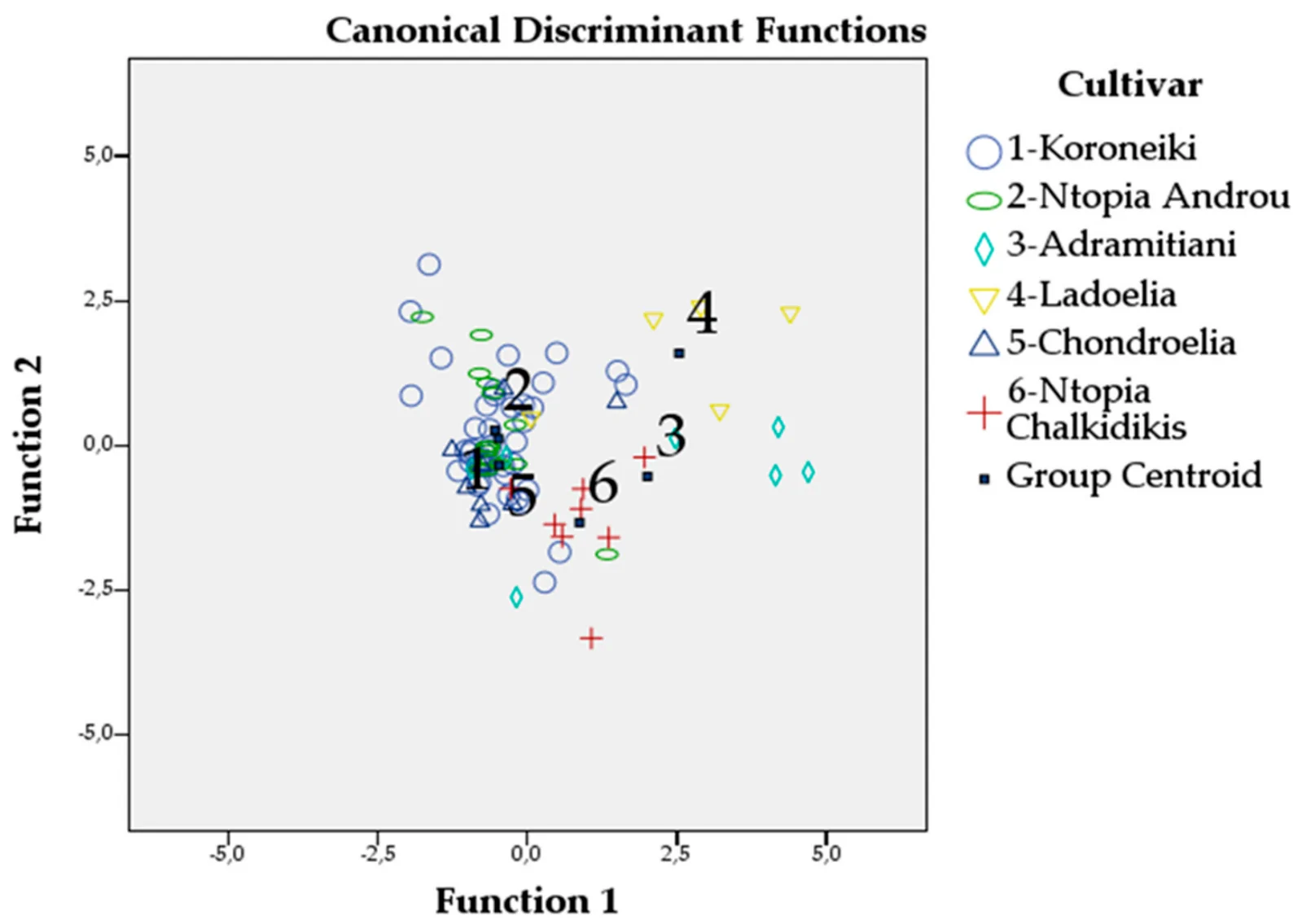
Classification of olive oil of the studied olive cultivars based on the significant minerals and LDA (considering that all groups are equal).

The overall correct classification rate was 62.5% for the original and 50% for the cross-validation method. The group centroid values were (−0.484, 0.117), (−0.545, 0.264), (2.007, −0.535), (2.538, 1.593), (−0.471, −0.347), and (0.872, −1.332) for olive oil samples from Koroneiki, Ntopia Androu, Adramitiani, Ladoelia, Chondroelia, and Ntopia Chalkidikis, respectively (Figure 7).

The best cross-validation results were obtained for olive oil samples from the Chondroelia cultivar (62.5%) (5/8 samples), followed by Koroneiki (55.6%) (25/45 samples) and Ntopia Chalkidikis (50%) (4/8 samples).

**Figure 8:**
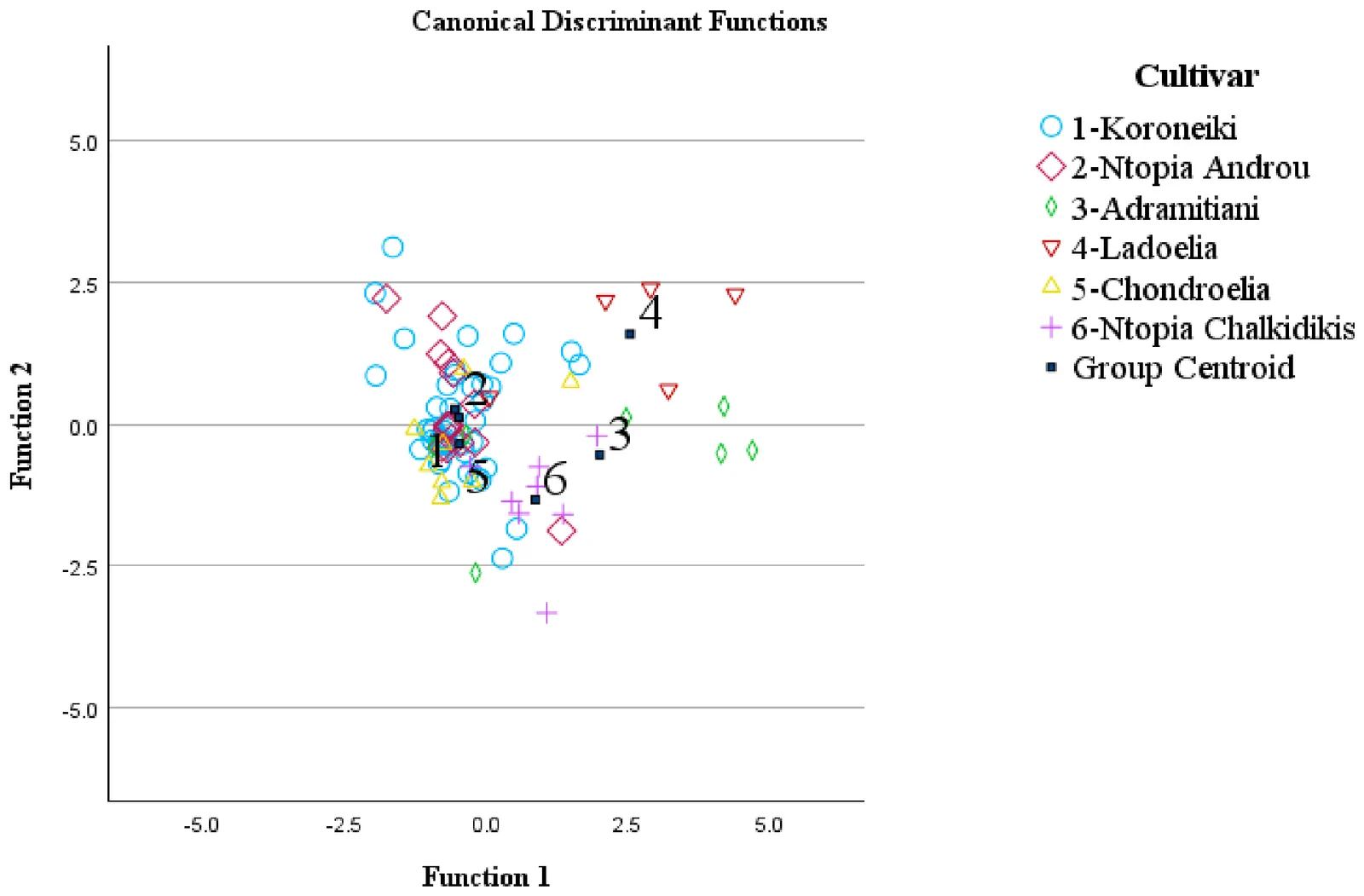
Classification of olive oil of the studied olive cultivars based on the significant minerals and LDA (compute from group sizes).

Taking into account the effect of the group sizes, LDA was run again. In this case, the overall correct classification rate was 67% for the original and 56.8% for the cross-validation method (Figure 8).

The best cross-validation results were obtained for olive oil samples from the Koroneiki cultivar (82.2%) (37/45) samples), followed by those of the Ladoelia cultivar (40%) (2/5) (Supplementary Table S7).

## 7. Discussion

The results of the present study are consistent with previous studies demonstrating that the geographical origin of olive oil influences its mineral profile. Dağdelen et al. [31] analyzed olive oil samples from different geographical regions of Turkey (Edincik, Gomec, Izmir) using ICP-OES and determined the concentrations of Fe, Mg, Sn, K and Ca. Detectable concentrations of the analyzed minerals were found in samples from the Gomec region, whereas no detectable concentrations were reported in samples from Izmir. These findings suggest that geographical origin and local soil conditions have an important role in the accumulation of minerals from the soil to the olive fruit and ultimately to the produced olive oil. The mineral profile observed in olive oils from the Gomec region is in agreement with the findings of the present study. This similarity may be associated with the geographical proximity of both regions to the Aegean Sea, as well as with comparable climatic and soil characteristics. Similarly, Bechar et al. [2] analyzed olive oil samples collected from four cities of the Fes-Meknes region in Morocco and reported variations in the concentration of As, Cr, Cd, Pb, Ni, Zn and Cu using ICP-OES. Potentially toxic elements were detected in some samples, which the authors attributed to local sources of environmental contamination. In contrast, toxic minerals were not detected in the majority of the samples analyzed in the present study, while Cr, Zn, Pb and Cu were detected only at very low concentrations. This difference suggests that the studied regions are subject to lower levels of environmental contamination by toxic elements. Likewise, Telloli et al. [32] investigated olive oil samples from different geographical regions of Italy and Albania using ICP-MS and reported the presence of Fe, P, Ca, Al and K. These elements showed similar concentrations among samples originating from Central Italy, whereas differences were observed in samples collected from Albania. The authors concluded that geographical origin plays a significant role in the mineral profile of olive oil, even when similar production and processing methods are applied. Finally, Zeiner et al. [33] analyzed olive oil samples from different regions of Croatia using ICP-AES and concluded that Al, Ca, Co, Cu, K, Mn and Ni could be used as geographical differentiation indicators. This finding is in agreement with the results of the present study, where significant regional differences were also observed for several minerals, supporting their potential use as indicators of geographical origin.

The classification of olive oils according to olive cultivar is particularly challenging, since the same cultivar may be grown in different regions and may additionally have different names depending on the local toponymy. Because of this, clear discrimination between olive cultivars is complex [34]. However, research on olives has shown that there are differences in their elemental profiles, a fact that supports the possibility of using specific minerals as indicators of olive cultivar. More specifically, a study conducted by Sahan et al. [35] showed that the mineral content of olives is influenced by both the stage of ripeness and the processing method. The above findings indicate that olive cultivar is a factor influencing the mineral profile of the resulting olive oil. Similar conclusions were reached in a study conducted on various Turkish olive cultivars, in which the following minerals K, P, Ca, Na, Mg, Fe, B, Cu, Li, P, Zn and Mn were determined using the ICP-AES method. The Gemlik cultivar exhibited a higher mineral content compared to the other cultivars (Kilis, Uslu, Turilye, Ayvalik) [36]. These findings are in agreement with those of the present study, where higher concentrations were found in the Ladoelia cultivar compared to the other cultivars.

We should also not forget that studies examining the mineral profile of olive oil from different geographical regions and olive cultivars in Greece are limited [15]. Comparing these previous studies makes it clear that both the geographical origin, including climate and soil conditions, along with the olive cultivar and production practices, affect the mineral profile of olive oil. Especially in the case of distinguishing the olive oil samples according to olive cultivar, the results in total were not satisfactory, probably for the reasons we mentioned before. This was not the case for the geographical origin classification of olive oil samples, where more satisfactory results were obtained.

In a previous study, Sayago et al. [37] performed mineral analysis in olive oil samples from different geographical regions of Spain characterized by Oceanic and Mediterranean climates. A total of 55 elements were determined in 125 samples using the ICP-MS/OES technique. Multivariate data analysis, such as partial least squares discriminant analysis (PLS-DA), was applied. The results demonstrated that the elemental fingerprint of the olive oils enabled discrimination between the Atlantic coast and Mediterranean coast geographical groups, with a classification accuracy of 92–97%. In another study, Benincasa et al. [38] examined the mineral profile of 36 olive oil samples from four different Italian regions representing two cultivars (Carolea and Coratina). Eighteen minerals were determined using ICP-MS, and LDA was applied for statistical analysis. Regarding the botanical origin of olive oil classification, the two cultivars did not completely separate. In contrast, geographical classification showed high discriminant power, as indicated by a Wilks’ lambda value close to 0. Additionally, another study investigated olive oil samples from four different regions of Spain and three different cultivars. Their mineral profile was analyzed using ICP-MS, and stepwise linear discriminant analysis (SLDA) was applied for statistical analysis. An overall classification accuracy of 93% was achieved for the geographical classification [39].

Taking into account the abovementioned and the present results, the mineral profile of olive oil may serve as a useful tool for the geographical origin classification of olive oil and additionally may show the purity of olive cultivars grown in a specific region.

## 8. Conclusions, Limitations, and Future Perspectives

This study investigated the potential of identifying the geographical and botanical origin of Greek olive oil samples based on their mineral profile using ICP-MS. The quality and composition of each olive oil depend on various factors, such as the olive variety, altitude, harvest year, extraction techniques, agricultural practices, processing technology, and geographical region. The results showed that it is possible to identify the geographical origin of olive oil more accurately based on its mineral profile, as pronounced differences were observed between regions. More extensively, the olive oil samples were satisfactorily classified, demonstrating that geographical origin, in combination with the soil differences in each region, has a significant effect on the mineral profile of the olive oil and especially Mn, Zn, Cu, Mg, Ti, Ba, Ga, Sr, Fe, and Rb. This contradicts the classification of olive oil samples according to olive cultivar. In the botanical origin analysis, olive oil samples of the studied cultivars showed low discriminatory power among cultivars, which can be attributed to the fact that some cultivars are identical but are referred to by the local producers under different names. However, some considerable differences were observed in the mineral content of the Ladoelia olive cultivar, which exhibited the highest concentrations of minerals. In addition, mineral content data for olive oil of the Ntopia Androu olive cultivar are reported for the first time in the literature. The results of this study demonstrate the potential of mineral element analysis, in combination with chemometric analysis, for characterizing the geographical variation of Greek olive oils. Analysis of the elemental profile of olive oils could be used as a complementary tool for assessing their authenticity based on geographical origin, particularly when the mineral profile does not correspond to the declared region. The classification performance of the linear discriminant analysis model was slightly affected (76.9% vs. 74.4%) when using the two prior probabilities mentioned before to establish representative mineral profiles for each geographical region. The same effect was observed for the cultivar origin classification of olive oil samples to a greater extent: a classification rate of 50% when using the hypothesis that all groups are equal and 56.8% when using the hypothesis compute from group sizes. Nevertheless, mineral content analysis of olive oil can serve as an effective tool for supporting its geographical and botanical origin authentication and safeguarding quality and traceability, while further research is needed concerning the botanical origin classification of olive oils studied in the present research. More specifically, the use of a larger number of olive oil samples, combined with chemometric methods and external evaluation of the statistical analysis data, along with more sophisticated techniques such as molecular analysis, could further strengthen the present findings and probably improve the discrimination rates of olive oils according to olive cultivar or geographical origin.

## Figures and Tables

**Table 1:** Mineral content of olive oil digest solutions according to geographical origin (μg/L).

	**Li**	**B**	**Na**	**Mg**	**Al**	**Ca**	**Ti**	**Cr**	**Mn**	**Fe**	**Ni**
**Aitoloakarnania**	0.17 ± 0.35 ^a^	0.28 ± 0.62 ^a^	1.74 ± 2.67 ^a^	1.24 ± 1.35 ^a^	9.62 ± 8.39 ^a^	1.81 ± 1.78 ^a^	0.05 ± 0.06 ^a^	0.07 ± 0.08 ^a^	0.01 ± 0.17 ^a^	1.48 ± 1.50 ^a^	0.02 ± 0.02 ^a^
**Andros**	0.15 ± 0.1 ^a^	0.00 ± 0.00 ^a^	0.74 ± 1.82 ^a^	0.16 ± 0.25 ^ab^	5.18 ± 4.50 ^a^	2.00± 1.83 ^ab^	0.00 ± 0.00 ^ab^	0.04 ± 0.02 ^a^	0.00 ± 0.00 ^a^	0.89 ± 0.50 ^a^	0.04 ± 0.07 ^a^
**Messinia**	0.27 ± 0.59 ^a^	0.00 ± 0.00 ^a^	0.67 ± 2.42 ^a^	0.05 ± 0.09 ^b^	3.52 ± 3.30 ^a^	0.01 ± 0.02 ^b^	0.00 ± 0.00 ^b^	0.05 ± 0.04 ^a^	0.00 ± 0.00 ^a^	0.44 ± 0.21 ^ab^	0.00 ± 0.00 ^ab^
**Fthiotida**	0.01 ± 0.00 ^a^	0.00 ± 0.00 ^a^	2.57 ± 0.72 ^a^	1.56 ± 0.87 ^a^	9.95 ± 4.35 ^a^	1.38 ± 0.48 ^ab^	0.02 ± 0.02 ^ab^	0.19 ± 0.01 ^a^	0.08 ± 0.04 ^b^	2.40 ± 0.59 ^ac^	0.07 ± 0.07 ^ac^
**Achaia**	0.01 ± 0.00 ^a^	0.00 ± 0.00 ^a^	0.32 ± 0.28 ^a^	1.04 ± 0.31 ^ab^	3.75 ± 0.94 ^a^	1.62 ± 2.80 ^ab^	0.00 ± 0.00 ^ab^	0.02 ± 0.02 ^a^	0.01 ± 0.04 ^a^	1.11 ± 0.11 ^a^	0.02 ± 0.03 ^a^
***p-*** **value**	0.726	0.293	0.411	0.003	0.058	0.018	0.010	0.394	0.000	0.006	0.015
	**Cu**	**Zn**	**Ga**	**Se**	**Rb**	**Sr**	**Zr**	**Mo**	**Ba**	**W**	**Pb**
**Aitoloakarnania**	0.17 ± 0.08 ^ac^	1.43 ± 0.92 ^a^	0.01 ± 0.02 ^a^	0.01 ± 0.01 ^a^	0.00 ± 0.00 ^ab^	0.07 ± 0.09 ^a^	0.02 ± 0.16 ^a^	0.04 ± 0.05 ^a^	0.08 ± 0.10 ^a^	0.21 ± 0.19 ^a^	0.02 ± 0.02 ^a^
**Andros**	0.09 ± 0.06 ^ab^	1.02 ± 1.08 ^ab^	0.01 ± 0.01 ^ab^	0.00 ± 0.00 ^a^	0.00 ± 0.00 ^a^	0.03 ± 0.04 ^ab^	0,01 ± 0.00 ^a^	0.09 ± 0.15 ^a^	0.04 ± 0.07 ^ab^	0.56 ± 0.80 ^a^	0.22 ± 0.35 ^a^
**Messinia**	0.02 ± 0.02 ^b^	0.30 ± 0.41 ^b^	0.00 ± 0.00 ^b^	0.00 ± 0.00 ^a^	0.00 ± 0.00 ^a^	0.00 ± 0.00 ^b^	0.00 ± 0.00 ^a^	0.15 ± 0.33 ^a^	0.00 ± 0.00 ^b^	0.63 ± 1.25 ^a^	0.00 ± 0.01 ^a^
**Fthiotida**	0.24 ± 0.06 ^c^	3.53 ± 1.57 ^c^	0.18 ± 0.01 ^ab^	0.00 ± 0.01 ^a^	0.00 ± 0.00 ^b^	0.10 ± 0.03 ^a^	0.01 ± 0.00 ^a^	0.00 ± 0.00 ^a^	0.13 ± 0.04 ^a^	0.04 ± 0.02 ^a^	0.07 ± 0.03 ^b^
**Achaia**	0.20 ± 0.03 ^ac^	0.40 ± 0.34 ^ab^	0.01 ± 0.00 ^ab^	0.00 ± 0.00 ^a^	0.00 ± 0.00 ^ab^	0.05 ± 0.00 ^ab^	0.02 ± 0.02 ^a^	0.00 ± 0.00 ^a^	0.06 ± 0.02 ^ab^	0.03 ± 0.03 ^a^	0.02 ± 0.00 ^a^
***p*** **-value**	0.000	0.000	0.028	0.373	0.008	0.019	0.087	0.513	0.009	0.437	0.000

Different letters in each column indicate statistically significant differences at the confidence level *p* < 0.05 according to Tukey’s honestly significant test (HSD).

**Table 2:** Mineral content of olive oil digest solutions according to olive cultivar (μg/L).

	Mn	Fe	Cu	Se	Rb	Na	Al	Ti	Ni
**Koroneiki**	0.01 ± 0.03 ^ab^	1.11 ± 1.09 ^a^	0.13 ± 0.10 ^a^	0.00 ± 0.01 ^ab^	0.00 ± 0.00 ^a^	1.19 ± 2.20 ^a^	6.52 ± 6.17 ^ab^	0.02 ± 0.05 ^ab^	0.02 ± 0.04 ^a^
**Ntopia Androu**	0.00 ± 0.00 ^a^	1.19 ± 0.61 ^a^	0.16 ± 0.17 ^ab^	0.00 ± 0.00 ^a^	0.00 ± 0.00 ^a^	0.12 ± 0.48 ^a^	8.74 ± 5.83 ^ab^	0.01 ± 0.01 ^a^	0.01 ± 0.02 ^a^
**Adramitiani**	0.04 ± 0.05 ^ab^	3.67 ± 3.89 ^b^	0.33 ± 0.31 ^b^	0.00 ± 0.01 ^ab^	0.00 ± 0.01 ^b^	5.43 ± 6.32 ^ab^	8.92 ± 6.95 ^ab^	0.03 ± 0.05 ^ab^	0.04 ± 0.05 ^ab^
**Ladoelia**	0.05 ± 0.06 ^b^	4.69 ± 3.29 ^bc^	0.36 ± 0.21 ^b^	0.00 ± 0.00 ^ab^	0.00 ± 0.00 ^ab^	8.03 ± 5.45 ^b^	13.39 ± 6.71 ^a^	0.07 ± 0.04 ^b^	0.07 ± 0.05 ^b^
**Chondroelia**	0.00 ± 0.01 ^ab^	1.21 ± 0.94 ^a^	0.13 ± 0.08 ^ab^	0.01 ± 0.01 ^b^	0.00 ± 0.00 ^a^	1.26 ± 1.89 ^a^	7.25 ± 5.61 ^ab^	0.02 ± 0.02 ^ab^	0.02 ± 0.01 ^ab^
**Ntopia Chalkidikis**	0.01 ± 0.03 ^ab^	1.56 ± 1.08 ^ab^	0.30 ± 0.11 ^b^	0.00 ± 0.00 ^ab^	0.00 ± 0.00 ^a^	1.16 ± 2.16 ^a^	2.87 ± 1.58 ^b^	0.00 ± 0.00 ^a^	0.03 ± 0.03 ^ab^
***p*** **-value**	0.129	0.000	0.017	0.060	0.000	0.873	0.131	0.107	0.368

Different letters in each column indicate statistically significant differences at the confidence level *p* < 0.05 according to Tukey’s honestly significant test (HSD).

**Table 3:** Analytical data of LDA for the discrimination of olive oil of the Koroneiki olive cultivar from Aitoloakarnania, Andros, Messinia, Fthiotida, and Achaia regions.

Test of Function(s)	Wilks’ Lambda	Chi-Square	df	*p*-Value	Eigenvalue	% of Variance	Cumulative %	Canonical Correlation
1 through 3	0.003	181.130	52	<0.001	11.970 ^a^	64.6	64.6	0.961
2 through 3	0.045	99.126	36	<0.001	4.383 ^a^	23.7	88.2	0.902
3 through 4	0.243	45.262	22	0.002	1.593 ^a^	8.6	96.8	0.784
4	0.630	14.766	10	0.141	0.586 ^a^	3.2	100.0	0.608

^a^. First 4 canonical discriminant functions were used in the analysis.

**Table 4:** Classification results of olive oil samples of the Koroneiki olive cultivar according to geographical origin.

LDA		Geographical Origin	Predicted Group Membership					Total
Method			Aitoloakarnania	Andros	Messinia	Fthiotida	Achaia	
Original ^a^	Count	Aitoloakarnania	13	1	0	0	1	15
		Andros	0	4	2	0	0	6
		Messinia	0	0	13	0	0	13
		Fthiotida	0	0	0	5	0	5
		Achaia	0	0	0	0	3	3
	%	Aitoloakarnania	86.7	6.7	0.0	0.0	6.7	100.0
		Andros	0.0	66.7	33.3	0.0	0.0	100.0
		Messinia	0.0	0.0	100.0	0.0	0.0	100.0
		Fthiotida	0.0	0.0	0.0	100.0	0.0	100.0
		Achaia	0.0	0.0	0.0	0.0	100.0	100.0
Cross-validated ^b,c^	Count	Aitoloakarnania	11	1	1	0	2	15
		Andros	1	1	4	0	0	6
		Messinia	1	0	12	0	0	13
		Fthiotida	0	0	0	4	1	5
		Achaia	2	0	0	0	1	3
	%	Aitoloakarnania	73.3	6.7	6.7	0.0	13.3	100.0
		Andros	16.7	16.7	66.7	0.0	0.0	100.0
		Messinia	7.7	0.0	92.3	0.0	0.0	100.0
		Fthiotida	0.0	0.0	0.0	80.0	20.0	100.0
		Achaia	66.7	0.0	0.0	0.0	33.3	100.0

a. In total, 90.5% of the original method’s grouped cases were correctly classified. b. Cross-validation is performed only for those cases in the analysis. In the cross-validation method, each case is classified by the functions derived from all cases other than that particular case. c. In total, 69.0% of the cross-validated method grouped cases were correctly classified.

**Table 5:** Contribution of the significant minerals for the classification of olive oil of the Koroneiki cultivar from Aitoloakarnania, Andros, Messinia, Fthiotida, and Achaia regions using LDA.

Structure Matrix				
Minerals	Discriminant Function			
	1	2	3	4
Mn	0.451 *	0.211	0.102	0.043
Cu	0.182	0.570 *	−0.083	0.259
Mg	0.086	0.318 *	0.042	−0.076
Ti	−0.035	0.264 *	0.239	−0.111
Ba	0.107	0.245 *	0.056	0.174
Sr	0.089	0.243 *	0.058	0.109
Fe	0.128	0.240 *	0.088	0.130
Ga	0.073	0.233 *	0.117	0.130
Rb	0.136	0.198 *	0.164	−0.060
Ca	−0.006	0.228	−0.018	0.492 **
Ni	0.144	0.087	0.023	0.423 **
Pb	0.250	0.206	0.083	0.364 **
Zn	0.266	0.268	0.281	0.335 **

Pooled within-group correlations between discriminating variables and standardized canonical discriminant functions. Variables ordered by absolute size of correlation within function. * Largest absolute correlation between each variable and any discriminant function. ** Non-significant discriminant function.

**Table 6:** Analytical data of LDA for the classification of olive oil of the Koroneiki olive cultivar from Aitoloakarnania, Andros, Messinia, and Fthiotida regions.

Test of Function(s)	Wilks’ Lambda	Chi-Square	df	*p*-Value	Eigenvalue	% of Variance	Cumulative %	Canonical Correlation
1 through 3	0.008	144.239	39	<0.001	12.959 ^a^	71.0	71.0	0.964
2 through 3	0.105	66.474	24	<0.001	4.602 ^a^	25.2	96.2	0.906
3	0.588	15.644	11	0.155	0.699 ^a^	3.8	100.0	0.642

^a^. First 3 canonical discriminant functions were used in the analysis.

**Table 7:** Contribution of the significant minerals for the classification of olive oil of the Koroneiki cultivar from Aitoloakarnania, Andros, Messinia, and Fthiotida regions using LDA.

Structure Matrix			
Minerals	Discriminant Function		
	1	2	3
Mn	0.443 *	0.161	−0.049
Zn	0.270 *	0.267	0.195
Cu	0.201	0.518 *	0.177
Mg	0.096	0.295 *	−0.118
Ti	−0.023	0.282 *	−0.171
Ba	0.114	0.232 *	0.111
Ga	0.081	0.230 *	0.064
Sr	0.096	0.230 *	0.054
Fe	0.133	0.227 *	0.063
Rb	0.139	0.192 *	−0.122
Ca	0.008	0.253	0.478 **
Ni	0.146	0.079	0.361 **
Pb	0.251	0.185	0.267 **

Pooled within-group correlations between discriminating variables and standardized canonical discriminant functions. Variables are ordered by the absolute size of correlation within the function. * Largest absolute correlation between each variable and any discriminant function. ** Non-significant discriminant function.

**Table 8:** Classification results of olive oil samples of the Koroneiki olive cultivar according to geographical origin, considering the ungrouped cases.

LDA		Geographical Origin	Predicted Group Membership				Total
Method			Aitoloakarnania	Andros	Messinia	Fthiotida	
Original ^a^	Count	Aitoloakarnania	14	1	0	0	15
		Andros	0	5	1	0	6
		Messinia	0	0	13	0	13
		Fthiotida	0	0	0	5	5
		Ungrouped cases	2	0	1	0	3
	%	Aitoloakarnania	93.3	6.7	0.0	0.0	100.0
		Andros	0.0	83.3	16.7	0.0	100.0
		Messinia	0.0	0.0	100.0	0.0	100.0
		Fthiotida	0.0	0.0	0.0	100.0	100.0
		Ungrouped cases	66.7	0.0	33.3	0.0	100.0
Cross-validated ^b,c^	Count	Aitoloakarnania	11	2	1	1	15
		Andros	2	2	2	0	6
		Messinia	0	0	13	0	13
		Fthiotida	1	0	0	4	5
	%	Aitoloakarnania	73.3	13.3	6.7	6.7	100.0
		Andros	33.3	33.3	33.3	0.0	100.0
		Messinia	0.0	0.0	100.0	0.0	100.0
		Fthiotida	20.0	0.0	0.0	80.0	100.0

a. In total, 94.9% of the original method grouped cases were correctly classified. b. Cross-validation is performed only for those cases in the analysis. In cross-validation, each case is classified by the functions derived from all cases other than that particular case. c. In total, 76.9% of the cross-validated grouped cases were correctly classified.

**Table 9:** Analytical data of LDA for the classification of olive oil from the different olive cultivars studied.

Test of Function(s)	Wilks’ Lambda	Chi-Square	df	*p*-Value	Eigenvalue	% of Variance	Cumulative %	Canonical Correlation
1 through 5	0.229	117.156	45	<0.001	1.015 ^a^	53.8	53.8	0.710
2 through 5	0.462	61.440	32	0.001	0.384 ^a^	20.4	74.2	0.527
3 through 5	0.639	35.593	21	0.024	0.240 ^a^	12.7	86.9	0.440
4 through 5	0.793	18.467	12	0.102	0.153 ^a^	8.1	95.0	0.365
5	0.914	7.129	5	0.211	0.094 ^a^	5.0	100.0	0.293

^a^. First 5 canonical discriminant functions were used in the analysis.

## Data Availability

The original contributions presented in this study are included in the article/Supplementary Material. Further inquiries can be directed to the corresponding author.

## Supplementary Materials

The following supporting information can be downloaded at: https://www.mdpi.com/article/10.3390/foods15193536/s1, Table S1: Meteorological stations, topography, and climate conditions of the studied regions, Table S2: Climate conditions of the studied regions of olive oil production during the harvesting years 2024 and 2025, Table S3: Operational conditions of the ICP-MS instrument, Table S4 Measured recovery rates, Table S5: Values of limits of detection and quantification and the correlation coefficient *R* of each calibration curve, Table S6: Geographical origin classification of olive oil of the Koroneiki olive cultivar using LDA and considering the effect size, Table S7: Cultivar origin classification of olive oil using LDA and considering the effect size, Figure 1: Calibration curves of the analyzed elements.
