# Supplementary material for: Botanical and Geographical Origin Assessment of Olive Oil from Known and Lesser-Known Greek Olive Cultivars Using Mineral Content and Chemometrics
Source: Foods. 2026 Oct 3;15(19):3536. doi: 10.3390/foods15193536 (PMC13665246; doi:10.3390/foods15193536)
Supplement: Supplementary file 1 [file foods-15-03536-s001.zip › foods-4439012-supplementary.pdf]

Supplementary material

Supplementary Table S1. Meteorological stations, topography, and climate conditions of the studied regions [29].

Stations	Potion	Altitude (m)	Sensor Height (m)	
			Temperature/Humidity*	Wind**
Andros	Gayrio Port	16	2	5
Messinia	Sailing Club	5	2	5
Aitoloakarnania	Department of Sustainable Agriculture/ University of Patras-Agrinio	72	2	2
Achaia	University of Patras	66	3	4
Lesvos	Agia Paraskevi	92	2	5
Fthiotida	Zeli	501	2	5
Chalkidiki	Traffic education park	580	2	5

* Height of temperature and humidity sensors; **Wind gauge height.

Supplementary Table S2. Climate conditions of the studied regions of olive oil production during the harvesting years 2024 and 2025 [29].

	Temperature (°C)	Wind Speed (km/h)	Rain (mm)	RH (min)	RH (max)
		**November 2024**			
Andros	16.1	14.9	50.4	59.4	78.7
Aitoloakarnania	13.9	3.0	164.8	54.9	82.8
Messinia	17.2	5.4	134.2	51.0	75.9
Achaia	12.4	2.1	107.2	1.1	174.3
Lesvos	12.7	4.8	60.4	58.0	84.0
Fthiotida	10.8	3.4	71.4	63.4	89.5
Chalkidiki	9.9	7.7	124.4	57.6	83.2
		**December 2024**			
Andros	14.2	17.1	206.6	64.4	84.3
Aitoloakarnania	10.6	2.9	292.0	66.5	88.6
Messinia	14.0	5.2	164.0	59.2	80.7
Achaia	9.1	9.1	174.6	0	287.4
Lesvos	10.4	3.9	216.6	67.2	86.8

Fthiotida	8.3	3.9	139.6	73.9	91.0
Chalkidiki	7.0	10.2	201.6	70.0	88.9
**January 2025**					
Andros	14.3	11.5	50.8	68.2	85.4
Aitoloakarnania	11.3	2.6	101.8	67.9	90.1
Messinia	13.9	4.3	211.8	63.2	85.1
Achaia	9.7	11.9	80.0	0	268.7
Lesvos	10.8	3.0	51.6	68.8	88.1
Fthiotida	8.3	2.7	89.6	73.7	92.7
Chalkidiki	6.8	5.3	17.6	70.1	90.0
**February 2025**					
Andros	10.7	15.1	77.2	58.8	78
Aitoloakarnania	9.5	2.8	80.8	57.4	84.2
Messinia	12.1	5.8	93.6	55.0	78.0
Achaia	7.3	14.4	52.4	0	309.9
Lesvos	6.6	5.4	83.0	60.0	83.0
Fthiotida	5.1	3.1	38.6	79.9	93.9
Chalkidiki	3.6	6.4	29.0	73.3	88.3
**March 2025**					
Andros	15.4	14.1	40.0	57.1	80.6
Aitoloakarnania	14.1	2.2	74.8	45.5	83.9
Messinia	15.7	6.0	43.4	49.9	77.5
Achaia	11.2	10	43	0.5	221
Lesvos	13.1	3.9	37.2	49.4	82.8
Fthiotida	11.4	4.2	36.6	60.5	83.5
Chalkidiki	10..4	7.2	82.6	52.3	78.6
**April 2025**					
Andros	16.1	11.6	48.0	53.1	78.6
Aitoloakarnania	16.2	2.7	20.8	46.6	83.8
Messinia	16.9	5.7	32.4	51.0	81.9
Achaia	13.3	12.2	10.6	4.4	157.0
Lesvos	13.2	4.1	21	51.6	83.1

Fthiotida	12.0	4.0	50.0	65.3	89.4
Chalkidiki	11.7	6.7	47.2	51.9	80.6
			**May 2025**		
Andros	20.6	10.7	5.0	45.1	76.8
Aitoloakarnania	20.2	2.1	41.6	44.3	82.3
Messinia	21.1	6.1	14.4	47.0	81.0
Achaia	17.3	8.6	19.6	29.6	61.6
Lesvos	19.7	9.6	36.5	77.7	15
Fthiotida	17.6	4.1	35.4	54.7	83.8
Chalkidiki	16.3	7.6	63.2	47.8	77.0

RH relative humidity

Supplementary Table S3. Operational conditions of the ICP-MS instrument [30].

Parameter	Value
RF Power	1550 W
Sampling Depth	8.0 mm
Nebulizer Gas	1.05 L/min
Helium Cell Gas	4.3 mL/min
Energy Discrimination	3.0 V
S/C Temperature	2 °C

Supplementary Table S4. Measured recovery rates (reference material IRMM-804 Rice Flour) [30].

Element	Certified value (mg/kg)	Measured value (mg/kg)	%Recovery
**As**	0.049	0.050	102%

**Cd**	1.61	1.67	103%
**Cu**	2.74	2.99	109%
**Zn**	23.1	23.3	101%
**Mn**	34.2	34.0	99%
**Pb**	0.42	0.45	107%

Supplementary Table S5. Values of limits of detection (ppb) and quantification (ppb) and the correlation coefficient *R* of each calibration curve [30].

ELEMENT	[1]LOD	[2]LOQ	[3]*R*
^{7}Li	0.0210	0.0699	1.0000
^{9}Be	0.0426	0.1421	1.0000
^{11}B	0.3825	1.2750	1.0000
^{23}Na	0.4059	1.3530	1.0000
^{24}Mg	0.4932	1.6440	1.0000
^{27}Al	0.4891	1.6303	1.0000
^{31}P	0.6581	2.1937	1.0000
^{39}K	1.7610	5.8700	1.0000
^{44}Ca	0.0885	0.2951	1.0000
^{47}Ti	0.0069	0.0229	1.0000
^{51}V	0.0175	0.0585	0.9999
^{52}Cr	0.0050	0.0167	0.9999
^{55}Mn	0.0886	0.2953	0.9999
^{56}Fe	0.0012	0.0041	0.9999
^{59}Co	0.0090	0.0299	1.0000
^{60}Ni	0.0064	0.0212	0.9999
^{63}Cu	0.1018	0.3393	0.9998
^{66}Zn	0.0094	0.0314	1.0000
^{69}Ga	0.0001	0.0003	0.9999
^{72}Ge	0.0020	0.0068	1.0000
^{75}As	0.0335	0.1117	1.0000
^{78}Se	0.0048	0.0159	1.0000
^{85}Rb	0.0066	0.0220	0.9999
^{88}Sr	0.0048	0.0161	0.9999
^{90}Zr	0.0018	0.0061	1.0000
^{93}Nb	0.0062	0.0208	1.0000
^{95}Mo	0.0019	0.0065	1.0000
^{107}Ag	0.0125	0.0418	1.0000
^{111}Cd	0.0016	0.0054	1.0000

**^{133}Cs**	0.0095	0.0318	1.0000
**^{137}Ba**	0.0011	0.0037	1.0000
**^{181}Ta**	0.0011	0.0037	1.0000
**^{182}W**	0.0022	0.0073	1.0000
**^{185}Re**	0.0020	0.0067	1.0000
**^{205}Tl**	0.0055	0.0182	1.0000
**^{208}Pb**	0.0006	0.0021	1.0000
**^{238}U**	0.0210	0.0699	1.0000

[1]LOD: Limits of Detection; [2]LOQ: Limits of Quantitation; [3]*R*: represents the correlation coefficient.

Supplementary Table S6. Geographical origin classification of olive oil of the Koroneiki olive cultivar using LDA and considering the effect size (compute from group sizes) (number of samples).

**Classification Results[a,c]**							
			Predicted Group Membership				
		Geographical Origin	Aitoloakarnania	Andros	Messinia	Fthiotida	Total
Original	Count	Aitoloakarnania	13	2	0	0	15
		Andros	0	5	1	0	6
		Messinia	0	0	13	0	13
		Fthiotida	0	0	0	5	5
		Ungrouped cases	0	1	2	0	3
	%	Aitoloakarnania	86.7	13.3	.0	.0	100.0
		Andros	.0	83.3	16.7	.0	100.0
		Messinia	.0	.0	100.0	.0	100.0
		Fthiotida	.0	.0	.0	100.0	100.0
		Ungrouped cases	.0	33.3	66.7	.0	100.0
Cross-validated[b]	Count	Aitoloakarnania	11	2	1	1	15
		Andros	2	1	3	0	6
		Messinia	0	0	13	0	13
		Fthiotida	1	0	0	4	5
	%	Aitoloakarnania	73.3	13.3	6.7	6.7	100.0
		Andros	33.3	16.7	50.0	.0	100.0
		Messinia	.0	.0	100.0	.0	100.0
		Fthiotida	20.0	.0	.0	80.0	100.0

a. 92.3% of original grouped cases were correctly classified.

b. Cross-validation is done only for those cases in the analysis. In the cross-validation method, each case is classified by the functions derived from all cases other than that particular case.

c. 74.4% of cross-validated method grouped cases correctly classified.

Supplementary Table S7. Cultivar origin classification of olive oil using LDA and considering the effect size (compute from group sizes) (number of samples).

Classification Results[a,c]									
			Predicted Group Membership						Total
		Cultivar Origin	Koroneiki	Ntopia Androu	Adramitiani	Ladoelia	Chondroelia	Ntopia Chalkidikis	
Original	Count	Koroneiki	38	2	0	2	2	1	45
		Ntopia Androu	8	6	0	0	0	1	15
		Adramitiani	2	0	3	1	1	0	7
		Ladoelia	1	0	0	4	0	0	5
		Chondroelia	5	1	0	0	2	0	8
		Ntopia Chalkidikis	2	0	0	0	0	6	8
	%	Koroneiki	84.4	4.4	.0	4.4	4.4	2.2	100.0
		Ntopia Androu	53.3	40.0	.0	.0	.0	6.7	100.0
		Adramitiani	28.6	.0	42.9	14.3	14.3	.0	100.0
		Ladoelia	20.0	.0	.0	80.0	.0	.0	100.0
		Chondroelia	62.5	12.5	.0	.0	25.0	.0	100.0
		Ntopia Chalkidikis	25.0	.0	.0	.0	.0	75.0	100.0
Cross-validated[b]	Count	Koroneiki	37	2	1	2	2	1	45
		Ntopia Androu	9	5	0	0	0	1	15
		Adramitiani	2	0	1	3	1	0	7
		Ladoelia	1	0	2	2	0	0	5
		Chondroelia	5	0	1	0	2	0	8
		Ntopia Chalkidikis	3	0	2	0	0	3	8
	%	Koroneiki	82.2	4.4	2.2	4.4	4.4	2.2	100.0
		Ntopia Androu	60.0	33.3	.0	.0	.0	6.7	100.0
		Adramitiani	28.6	.0	14.3	42.9	14.3	.0	100.0
		Ladoelia	20.0	.0	40.0	40.0	.0	.0	100.0
		Chondroelia	62.5	.0	12.5	.0	25.0	.0	100.0
		Ntopia Chalkidikis	37.5	.0	25.0	.0	.0	37.5	100.0
a. 67.0% of the original grouped cases were correctly classified.									
b. Cross-validation is done only for those cases in the analysis. In the cross-validation method, each case is classified by the functions derived from all cases other than that particular case.									
c. 56.8% of cross-validated method grouped cases were correctly classified.									

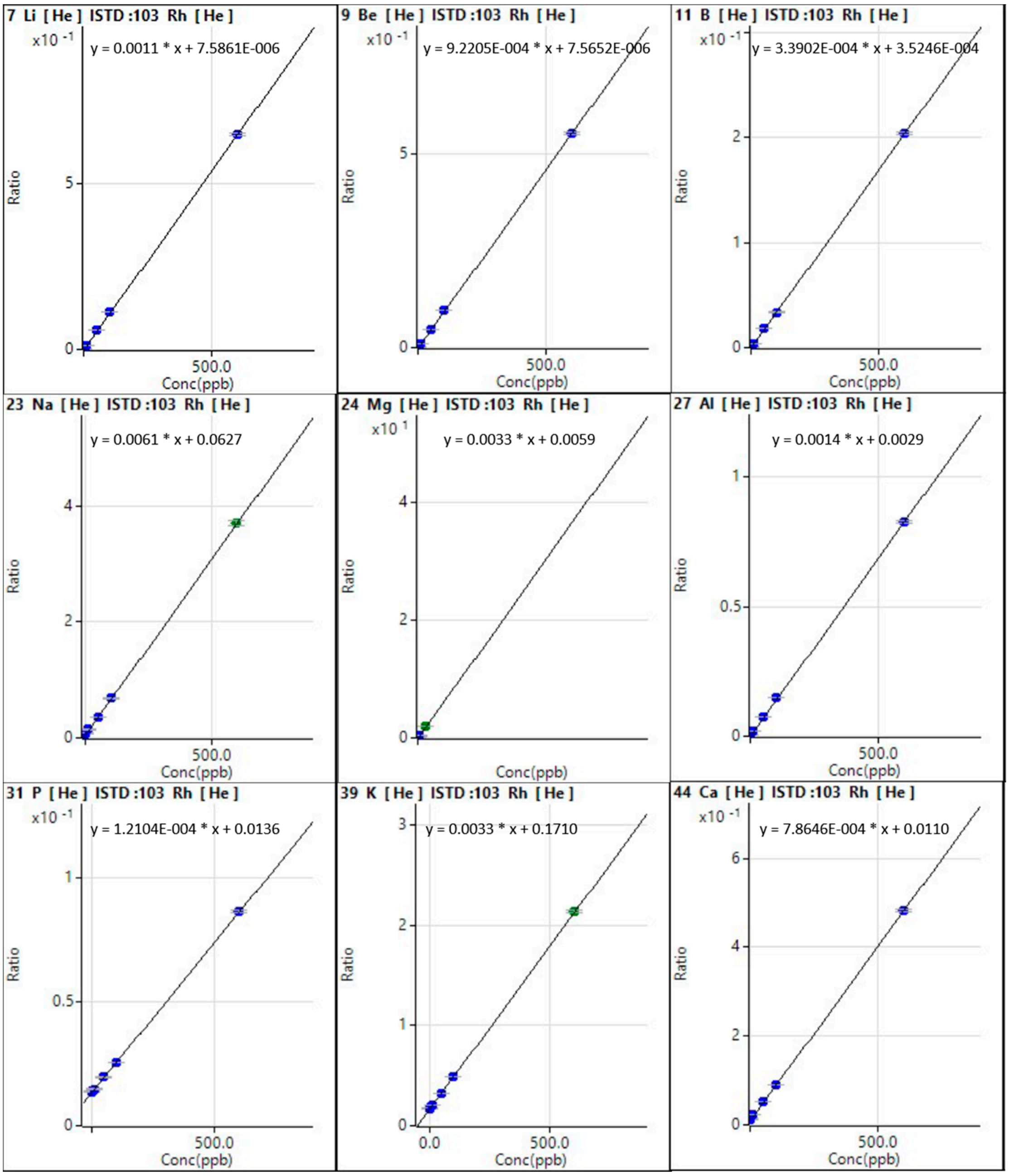

7 Li [He] ISTD :103 Rh [He]
y = 0.0011 * x + 7.5861E-006
9 Be [He] ISTD :103 Rh [He]
y = 9.2205E-004 * x + 7.5652E-006
11 B [He] ISTD :103 Rh [He]
y = 3.3902E-004 * x + 3.5246E-004
23 Na [He] ISTD :103 Rh [He]
y = 0.0061 * x + 0.0627
24 Mg [He] ISTD :103 Rh [He]
y = 0.0033 * x + 0.0059
27 Al [He] ISTD :103 Rh [He]
y = 0.0014 * x + 0.0029
31 P [He] ISTD :103 Rh [He]
y = 1.2104E-004 * x + 0.0136
39 K [He] ISTD :103 Rh [He]
y = 0.0033 * x + 0.1710
44 Ca [He] ISTD :103 Rh [He]
y = 7.8646E-004 * x + 0.0110
Ratio
Conc(ppb)

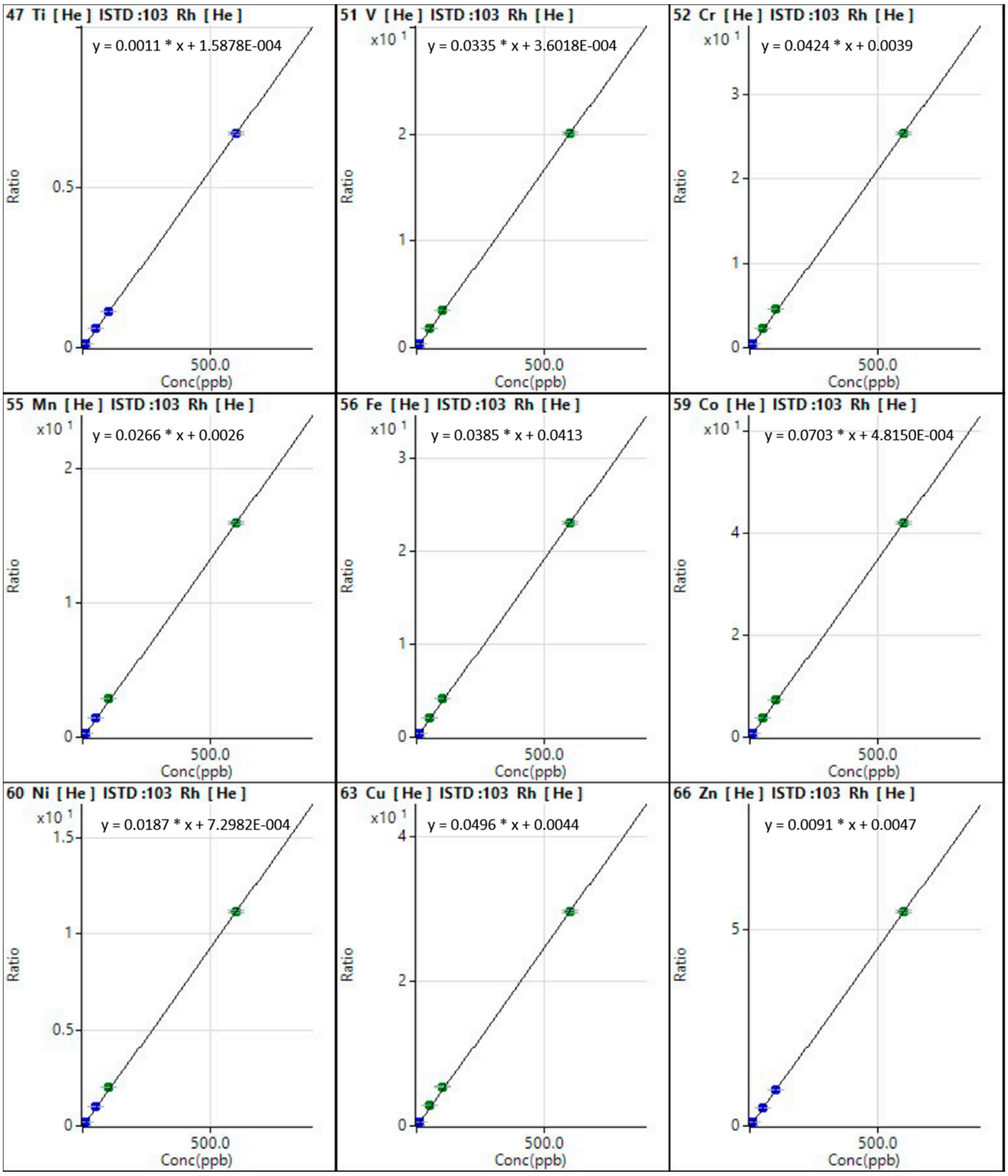
47 Ti [He] ISTD :103 Rh [He]
y = 0.0011 * x + 1.5878E-004
51 V [He] ISTD :103 Rh [He]
y = 0.0335 * x + 3.6018E-004
52 Cr [He] ISTD :103 Rh [He]
y = 0.0424 * x + 0.0039
55 Mn [He] ISTD :103 Rh [He]
y = 0.0266 * x + 0.0026
56 Fe [He] ISTD :103 Rh [He]
y = 0.0385 * x + 0.0413
59 Co [He] ISTD :103 Rh [He]
y = 0.0703 * x + 4.8150E-004
60 Ni [He] ISTD :103 Rh [He]
y = 0.0187 * x + 7.2982E-004
63 Cu [He] ISTD :103 Rh [He]
y = 0.0496 * x + 0.0044
66 Zn [He] ISTD :103 Rh [He]
y = 0.0091 * x + 0.0047
x10 1
Ratio
Conc(ppb)
500.0

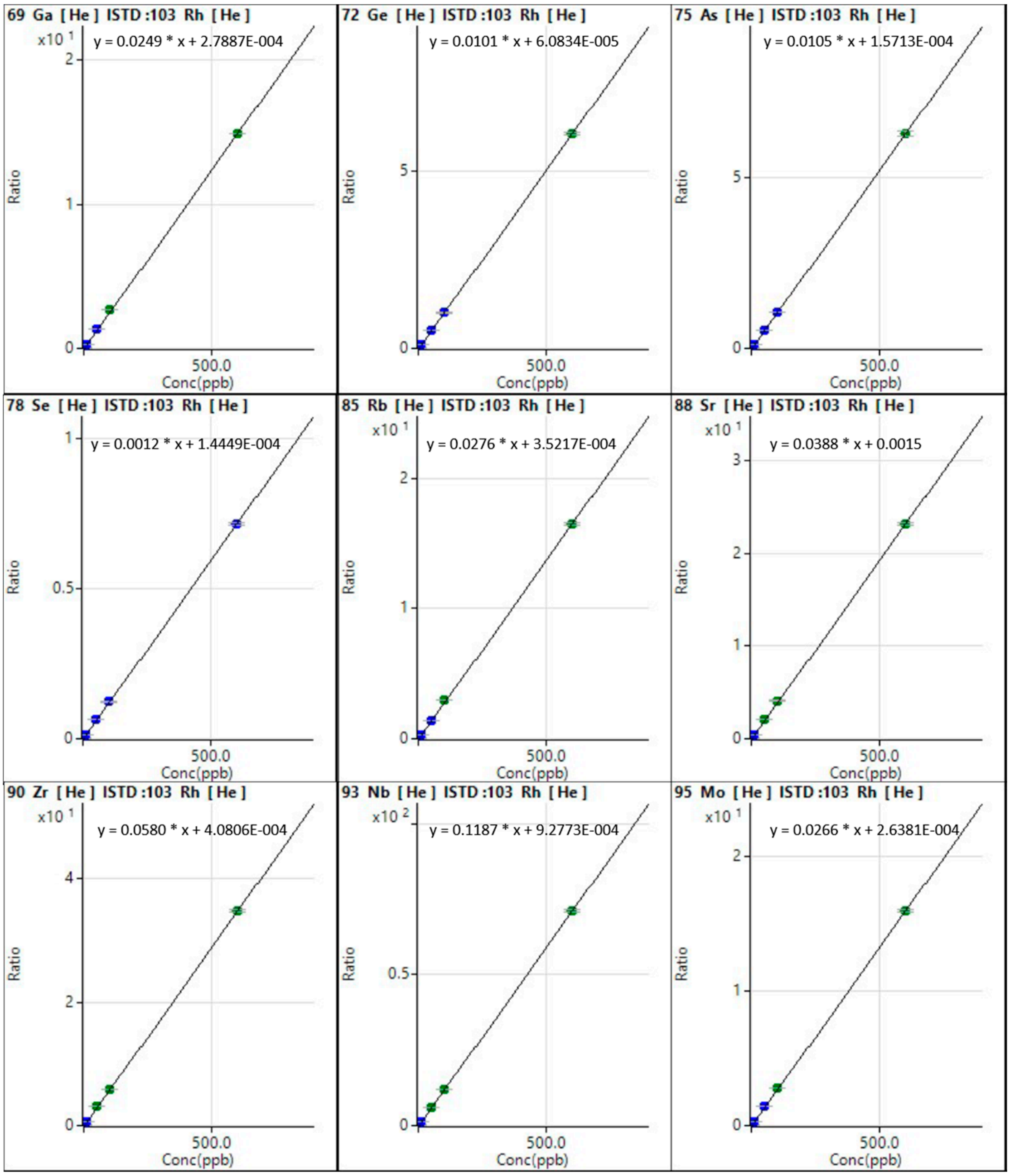

69 Ga [He] ISTD :103 Rh [He]
x10 1
y = 0.0249 * x + 2.7887E-004
Ratio
Conc(ppb)
72 Ge [He] ISTD :103 Rh [He]
y = 0.0101 * x + 6.0834E-005
Ratio
Conc(ppb)
75 As [He] ISTD :103 Rh [He]
y = 0.0105 * x + 1.5713E-004
Ratio
Conc(ppb)
78 Se [He] ISTD :103 Rh [He]
y = 0.0012 * x + 1.4449E-004
Ratio
Conc(ppb)
85 Rb [He] ISTD :103 Rh [He]
x10 1
y = 0.0276 * x + 3.5217E-004
Ratio
Conc(ppb)
88 Sr [He] ISTD :103 Rh [He]
x10 1
y = 0.0388 * x + 0.0015
Ratio
Conc(ppb)
90 Zr [He] ISTD :103 Rh [He]
x10 1
y = 0.0580 * x + 4.0806E-004
Ratio
Conc(ppb)
93 Nb [He] ISTD :103 Rh [He]
x10 2
y = 0.1187 * x + 9.2773E-004
Ratio
Conc(ppb)
95 Mo [He] ISTD :103 Rh [He]
x10 1
y = 0.0266 * x + 2.6381E-004
Ratio
Conc(ppb)

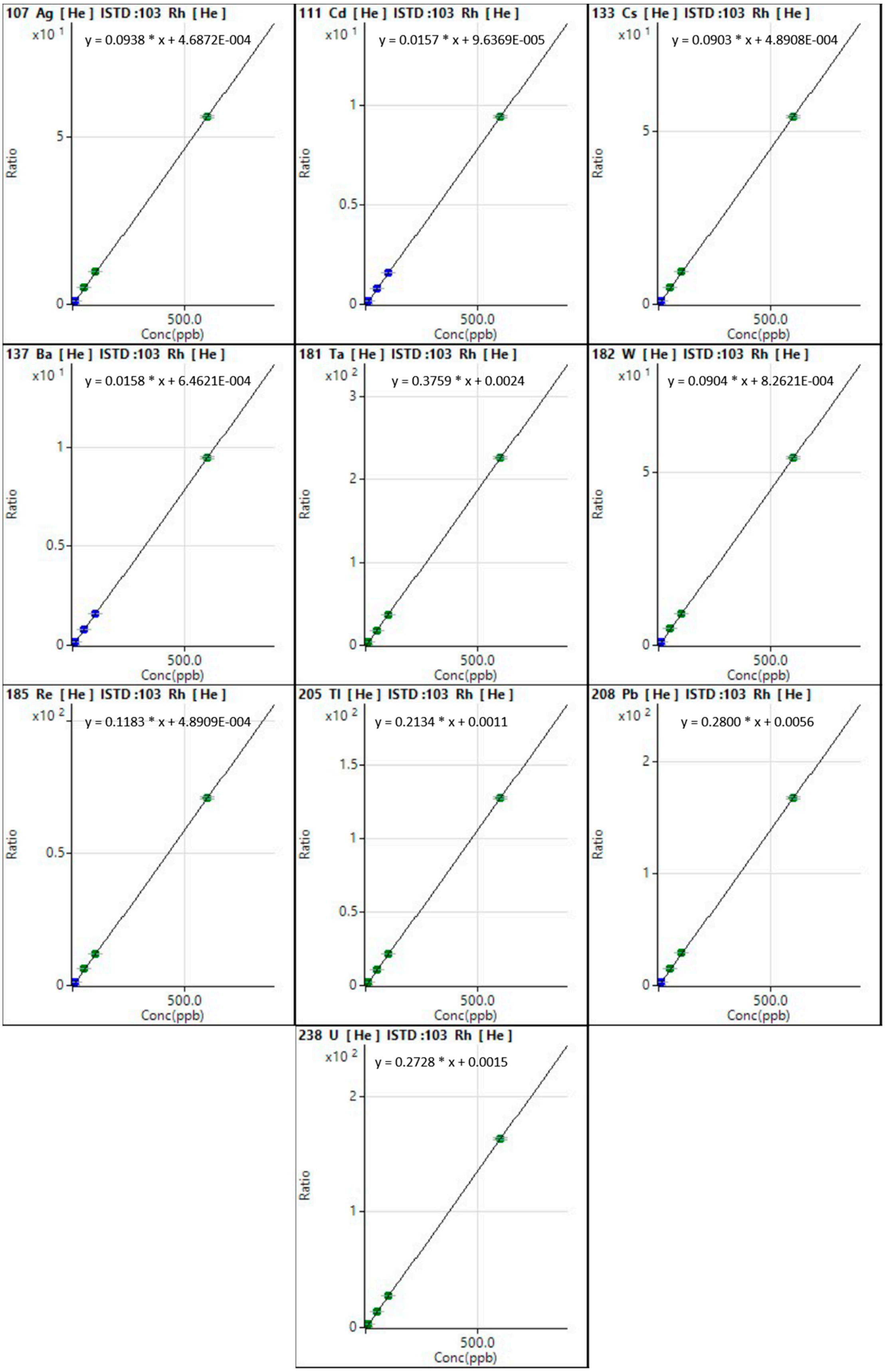

Figure S1. Calibration curves of the analyzed elements.
